# Multi-level 3D genome organization deteriorates during breast cancer progression

**DOI:** 10.1101/2023.11.26.568711

**Published:** 2023-11-27

**Authors:** Roberto Rossini, Mohammadsaleh Oshaghi, Maxim Nekrasov, Aurélie Bellanger, Renae Domaschenz, Yasmin Dijkwel, Mohamed Abdelhalim, Philippe Collas, David Tremethick, Jonas Paulsen

**Affiliations:** 1. Department of Biosciences, Faculty of Mathematics and Natural Sciences, University of Oslo, 0316 Oslo, Norway; 2. Department of Genome Sciences, The John Curtin School of Medical Research, The Australian National University, Canberra, Australian Capital Territory, Australia; 3. Department of Molecular Medicine, Institute of Basic Medical Sciences, Faculty of Medicine, University of Oslo, 0317 Oslo, Norway; 4. Department of Immunology and Transfusion Medicine, Oslo University Hospital, 0424 Oslo, Norway; 5. Centre for Bioinformatics, Department of Informatics, University of Oslo, 0316 Oslo, Norway

## Abstract

Breast cancer entails intricate alterations in genome organization and expression. However, how three-dimensional (3D) chromatin structure changes in the progression from a normal to a breast cancer malignant state remains unknown. To address this, we conducted an analysis combining Hi-C data with lamina-associated domains (LADs), epigenomic marks, and gene expression in an *in vitro* model of breast cancer progression. Our results reveal that while the fundamental properties of topologically associating domains (TADs) remain largely stable, significant changes occur in the organization of compartments and subcompartments. These changes are closely correlated with alterations in the expression of oncogenic genes. We also observe a restructuring of TAD-TAD interactions, coinciding with a loss of spatial compartmentalization and radial positioning of the 3D genome. Notably, we identify a previously unrecognized interchromosomal insertion event, wherein a locus on chromosome 8 housing the *MYC* oncogene is inserted into a highly active subcompartment on chromosome 10. This insertion leads to the formation of *de novo* enhancer contacts and activation of the oncogene, illustrating how structural variants can interact with the 3D genome to drive oncogenic states. In summary, our findings provide evidence for the degradation of genome organization at multiple scales during breast cancer progression revealing novel relationships between genome 3D structure and oncogenic processes.

## Introduction

The mammalian genome is folded into large-scale dynamic 3-dimensional (3D) chromatin conformations that provide the framework for regulated gene expression. In the interphase nucleus, chromosomes exist as distinct territories (Rowley and Corces 2018; Cremer and Cremer 2010) within which gene-rich and active A-compartments are segregated from more gene-poor and inactive B compartments (Misteli 2004; Crosetto and Bienko 2020; Lieberman-Aiden et al. 2009), both ranging in size from single promoters up to 2 megabase-pairs (Mbp) (Sood and Misteli 2022; Harris et al. 2023). Compartments consist of multiple sub-compartments that differ in their chromosomal contact frequencies, enrichment in histone modifications and chromatin-binding proteins (Spracklin et al. 2023). At a resolution ranging from ~10–800 kilobase-pairs (kbp), topologically associated domains (TADs) emerge as local, highly interacting domains delineated by boundaries enriched in CTCF (Rao et al. 2014; Nora et al. 2012; Dixon et al. 2012). TADs are further organized into sub-TADs and mediate the formation of internal smaller chromatin loops, which includes enhancer-promoter interactions (Berlivet et al. 2013). TADs and sub-TADs seemingly form independent of compartments (Rowley and Corces 2018). It was initially proposed that TADs provide a platform for the coordinated regulation of subsets of genes by enhancers located within the boundaries of the TAD. However, disrupting TAD structure minimally affects gene expression (Nora et al. 2017; Rao et al. 2017; Schwarzer et al. 2017), suggesting that other levels of genome organization have a more important regulatory role in interphase.

An intriguing possibility is that long-range TAD-TAD interactions, forming TAD cliques (Zhao et al. 2023; Pei et al. 2022; Li et al. 2022; Arnould et al. 2023; Paulsen et al. 2019), sculpt the 3D genome into distinct functional domains. TAD cliques are enriched in B compartments, and can gain or lose TADs during differentiation, generally in correlation with gene repression or activation, respectively (Paulsen et al. 2019). Additionally, the nuclear envelope imposes positional constraints on chromatin by anchoring primarily heterochromatin through lamina-associated domains (LADs) of ~0.1–10 Mbp in size (Gonzalez-Sandoval and Gasser 2016; Rønningen et al. 2015; van Steensel and Belmont 2017). Accordingly, genes located at the nuclear periphery are repressed or expressed at lower levels than genes localized towards the nuclear center (Misteli 2004; Crosetto and Bienko 2020). Interestingly, integrating Hi-C and LAD data allows the generation of 3D structural genome models that accurately recapitulate the radial nuclear positions of TADs (Paulsen et al. 2017, 2018; Li et al. 2017; Boninsegna et al. 2022), and provide an enhanced understanding of the link between changes in chromatin architecture and gene regulation upon onset of disease (Paulsen et al. 2017).

Indeed, an increased number of diseases, including cancer, have attributed transcriptional dysregulation to alterations in 3D genome organization (Feng and Pauklin 2020; Osman et al. 2022). Yet, Analysis of multiple cancers shows that TAD boundary deletions only rarely change gene expression, emphasizing their rare involvement in gene expression dysregulation in cancer (Akdemir et al. 2023). Even with significant shifts in A and B compartments between colon tumors and normal intestinal cells, TADs remain relatively unchanged (Johnstone et al. 2020), suggesting compartment switching significantly impacts gene expression control (Sood and Misteli 2022; Ibrahim and Mundlos 2020).

Breast cancer is the most common cancer in women (Bobbitt et al. 2023). These cancers have been classified into five subtypes based on the expression of receptors for human epidermal growth factor, estrogen and progestogen, and clinical features (Bobbitt et al. 2023). Among these subtypes, triple-negative breast cancers, which do not express any of these receptors, are the most aggressive (Bobbitt et al. 2023). Few studies have examined the chromatin architectural features of breast cancer cells or tissues (Dozmorov et al. 2023; Kim et al. 2022; Wang et al. 2022; Zhou et al. 2019). Nonetheless, one study reports that triple-negative breast cancer cells display the most severe disruption of the 3D genome, including weakening of TAD borders, loss of 3D chromatin interactions leading to fewer chromatin loops, and dynamic compartmental changes (Kim et al. 2022). However, how 3D chromatin organizational changes are associated with the progression from a normal-to a breast cancer malignant state has remained unexplored.

Here, we rely on an *in vitro* isogenic breast cancer progression model (Dawson et al. 1996; Santner et al. 2001) to investigate 3D genome organization changes in this process. We report a hitherto unrecognized multi-level reorganization of the genome consisting of (i) A/B compartment and subcompartment switching as a main feature linked to gene expression dysregulation in breast cancer; (ii) corresponding reconfiguration of TAD cliques; (iii) deterioration of radial sub-compartment organization; and (iv) a hitherto unreported inter-chromosomal insertion event placing the *MYC* locus proximal to active enhancers.

## Results

### TAD properties are conserved across breast cancer stages

We performed duplicate Hi-C experiments in three stages of a human breast cancer progression model, corresponding to a nonmalignant state (using MCF10A cells, “10A” from here on), a premalignant state (MCF10AT1 cells; “T1”) and a malignant tumorigenic state (MCF10Ca1a cells; “C1”). We obtained an average of >560 million pairwise interactions after filtering (Supplementary Table S1), supporting analyses in the range of 1–10 kbp bin resolution (Lajoie et al. 2015; Ay and Noble 2015). Filtering statistics and the relative fraction of intra- (cis) and inter- (trans) chromosomal contacts indicate high quality libraries (Lajoie et al. 2015), and samples show high reproducibility between replicates (Supplementary Fig. S1 and S2) (Yang et al. 2017). We identified and masked out regions with translocations and used ICE-normalized interactions which adjusts for bias resulting from copy-number alterations (see Methods)(Kim et al. 2022).

**Fig. 1.**
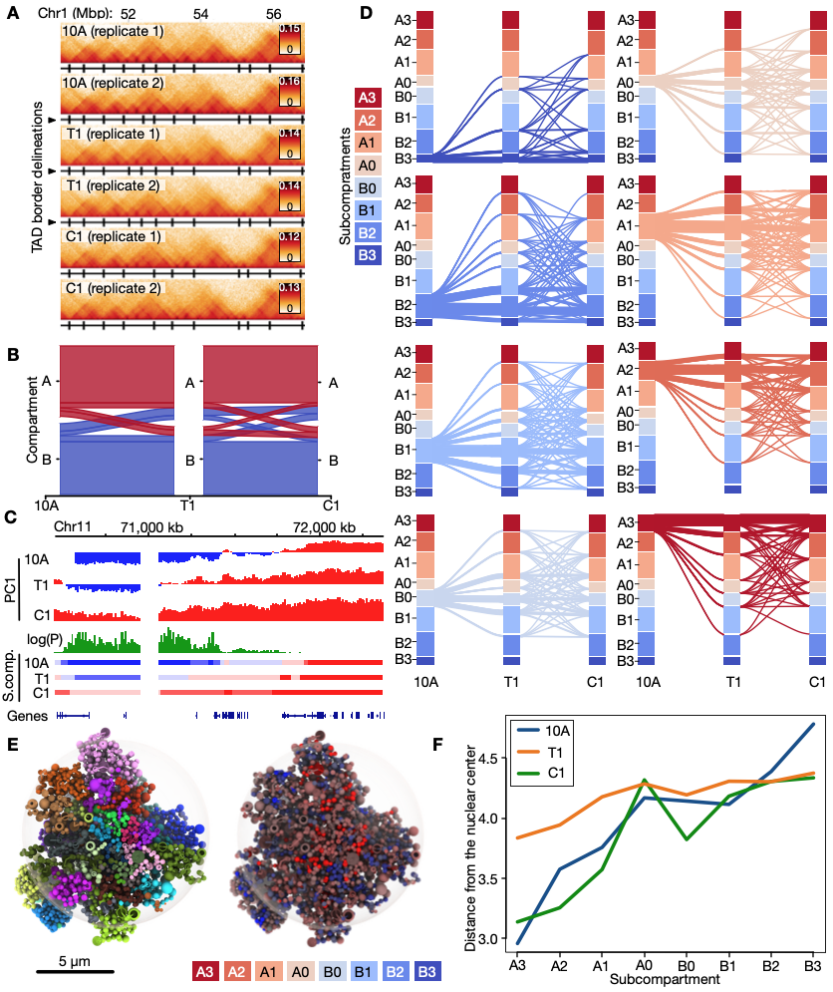
Alterations in genome compartmental properties during breast cancer progression stages. **A:** Example of Hi-C data for a region on chromosome 1. TAD delineations are shown as black vertical lines below each track. **B**: Alluvial plot showing A/B compartment conservation and switching during progression from 10A (left), via T1 (middle) and to C1 (right). **C**: Example region on chromosome 11 showing a statistically significant switch in the first principal component (PC1) in the three stages. Corresponding subcompartments in the three stages (10A, T1, C1) are shown below. **D**: Subcompartment switches across 10A, T1 and C1 shown as separate alluvial plots starting at each of the eight different subcompartments in 10A. **E**: Left: Tomographic view of exemplary Chrom3D model from 10A cells with chromosomes colored individually. Right: The same model with regions colored by their subcompartment associations. **F**: Plot of median distance from the nuclear center for each subcompartment in each condition.

TAD comparisons across samples and replicates (Fig. 1A) show similar TAD numbers and genomic size (Supplementary Fig. S3), TAD border insulation (Supplementary Fig. S4) and intra-TAD contact frequencies (Supplementary Fig. S5), and genomic positions of both TADs (50kb), and sub-TADs (10kb) (Supplementary Fig. S6). Thus, we conclude that TADs are overall similar across the three cell types and are therefore likely not the main oncogenic drivers in our progression model.

### Compartments and subcompartments engage in major switching events during breast cancer progression

To map differences in long-range genome contacts in our progression model, we searched for any identified A/B compartment switching, a property previously reported to be relevant for breast cancer (Barutcu et al. 2015). Most of the genome (~79%) remains in the same compartment across the progression stages (Fig. 1B). However, for the fraction that switches between states, we note 374 Mbp in 10A switching to either B (180 Mbp) or A (193 Mbp) compartments in T1 (Fig. 1B and Supplementary Table S2). Most of the switched compartments remain in their switched state in C1, but a fraction switches back to B (75 Mbp) or A (74 Mbp). Thus, compartment switching is a prominent feature in this breast cancer progression model.

We next used dcHiC to identify subcompartments (Fig. 1C) (Barutcu et al. 2015). We predict four A sub-compartments (A0-A3) and four B sub-compartments (B0-B3), with A1, A2 and B1 being generally the most prominent and A0 and B0 the least prominent for all cell stages (Supplementary Fig. S7; Supplementary Table S3). Overlaying a range of active and repressive histone modifications from public ChIP-seq datasets onto the subcompartments reveals a gradual enrichment of the ratio of heterochromatin to euchromatin features, going from B3 to A3 subcompartments (Supplementary Fig. S8). From this analysis, B2 is more reminiscent of constitutive heterochromatin, whereas B3 shows features or facultative heterochromatin based on the ratio of H3K9me3 to H3K27me3. Compared to the other subcompartments, A0 and B0 are the least enriched in active and repressive histone marks respectively, raising the interesting possibility that they could be more easily remodeled into other stronger euchromatic or heterochromatic subcompartments. In contrast, A3 and B3 display the strongest active and inactive states respectively, which would arguably be more resistant to configuration changes (Supplementary Fig. S8). Indeed, we find that (i) weaker, intermediate subcompartments (A0, A1, B0, B1) undergo more dynamic switches than more prominent and stronger subcompartments (A2, A3, B2, B3) (Fig. 1D; Supplementary Fig. S9–11). Yet, interestingly, (ii) switching between B0 and A0 (B0↔A0) is minimal; whereas (iii) B0↔A1 is the most prominent inter-compartment switch (Fig. 1D; Supplementary Fig. S9–11), indicating specific switching between weakly heterochromatic and euchromatic states. A further characterization of paths of subcompartment switching across the three stages shows that (iv) a similar fraction of 24–26% of subcompartments ends up in either a more closed (and thus more B-like) subcompartment in C1, or a more open (A-like) subcompartment, while reverting to the initial 10A-subcompartment landscape in C1 rarely happens (Supplementary Fig S12). Lastly, (v) switches predominantly involves a transition between consecutive subcompartment strengths, such as moving from B2 to B3, with essentially as frequent occurrences of shifts towards more open subcompartments (e.g., A2→A3) and less open ones (e.g., B2→B3) (see Supplementary Fig S12).

To determine implications of subcompartment switching on 3D genome organization, we generated 3D genome models (Paulsen et al. 2017) integrating Hi-C data with nuclear lamina-chromatin interactions (LADs) mapped by ChIP-seq of lamin B1 in each cell type (see Methods). The resulting models (Fig. 1E; Supplementary Fig. 13–15) display expected genomic features, with gene-rich and more gene-poor chromosomes located more frequently towards the nuclear interior or periphery, respectively (Supplementary Fig. S16–18). Strikingly, 3D modeling of all three cell types reveals increased nuclear peripheral localization of A3 to B3 subcompartments (Fig. 1F). Further, there is a more prominent radial segregation of subcompartments in 10A cells than in the T1 and C1 cancer cells (Fig. 1F). In fact, B2 and B3 subcompartments seem consistently shifted away from the nuclear periphery in the T1 and C1 cells, compared to 10A (Fig. 1F; Supplementary Fig S19; Supplementary Table S4), which concurs with a reduction in LAD coverage in these cells (Supplementary Fig. S20). Notably, T1 cells display the most dramatic change in radial subcompartment positioning compared to 10A and C1, with A1, A2 and A3 also being closer to the nuclear periphery (Fig. 1F). Taken together, our data indicate that subcompartment switching characterizing the 10A-T1-C1 transition is accompanied by a deterioration in radial disposition of peripheral heterochromatin in the breast cancer progression model.

### Subcompartment switching reflects changes in gene expression

**Fig. 2.**
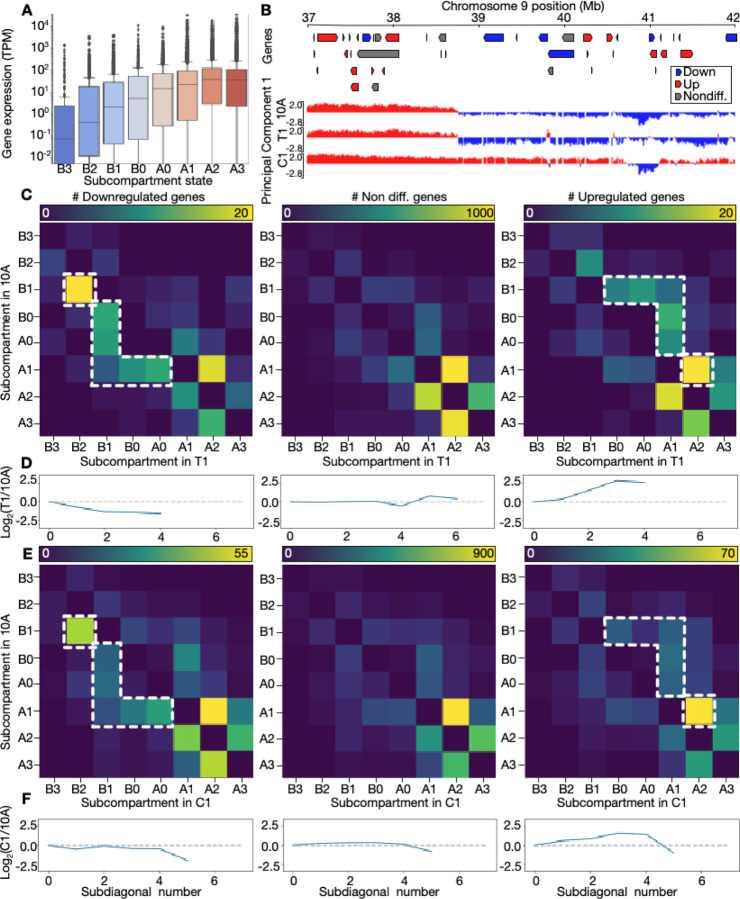
Coordinated changes in subcompartments and gene expression. **A**: Gene expression levels (TPM) by subcompartment state. Vertical axis in log scale. **B**: Example of subcompartment switching on chromosome 9. **C**: Heatmaps showing number of DE downregulated genes (left panel), non-differential genes (middle panel) and upregulated genes (right panel) in subcompartments switching between 10A (vertical axis) and T1 (horizontal axis). Dotted lines highlight regions with enrichment relative to non-differential and upregulated genes. **D**: Log2-ratio of number of genes in subdiagonal sums in the upper vs. lower triangular of the corresponding heatmap from C. **E**: Heatmaps as in C but contrasting 10A with C1. Dotted lines highlight regions with enrichment relative to non-differential and downregulated genes. **F**: Log2-ratio plots as in D, but contrasting 10A with C1.

Having discovered systematic genome structural changes in the breast cancer progression model, we next investigated whether this is linked to transcriptional changes by performing RNA-seq analyses. Differential analysis between the cell types reveals 3180 differentially expressed (DE) genes between 10A and T1 cells, and 8362 DE genes between 10A and C1 cells (Supplementary Table S5). Analysis of disease-linked gene set enrichment for DE genes in T1 and C1, reveals that “breast carcinoma” is the most significant associated disease term, followed by other cancer types. On the other hand, “organ system benign neoplasm” is the second most associated term for T1, whereas this term is absent for C1, reflecting the expected transcriptomic differences between the transformed (T1) versus the malignant (C1) cell line (Supplementary Fig. S21). Overlaying the RNA-seq data onto the matched Hi-C-based subcompartments in all three cancer progression stages, we observe the anticipated associations wherein transcription exhibits a gradual increase from B3 to A3 (Fig. 2A, Supplementary Fig. S22), reinforcing the validity of the subcompartment classification and the RNA-seq data in our samples.

**Fig. 3.**
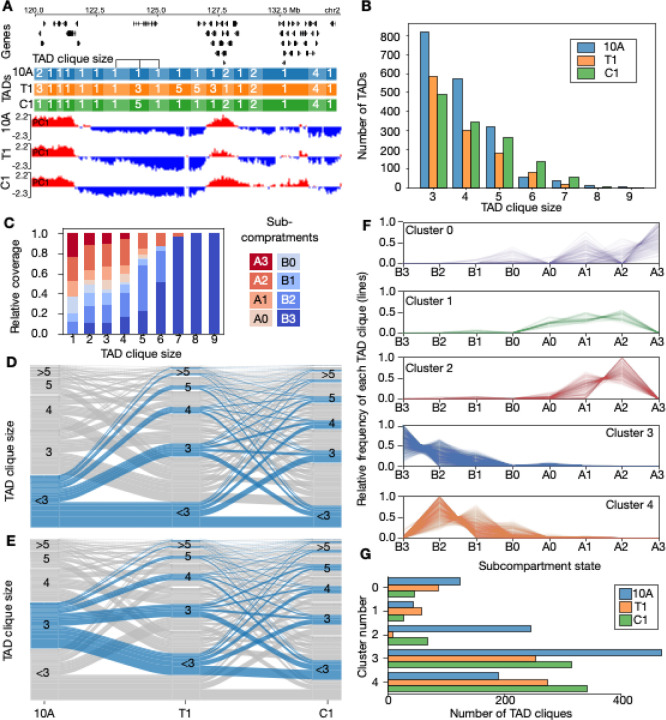
TAD clique dynamics during breast cancer progression stages. **A**: Example region on chromosome 2 showing changes in TAD clique sizes and compartment eigenvalues. **B**: Absolute number of TADs in cliques with maximal size ranging from 3 to 9 shown for each of the three breast cancer stages. **C**: Relative coverage of subcompartments for non-cliques and TAD cliques of size ranging from 3–9. **D**: Alluvial plots highlighting the alluvial path of a non-clique in 10A across the three cancer stages. **E**: Alluvial path of a TAD clique of size 3 in 10A. **F**: TAD clique clustering into 5 clusters based on their enrichment in subcompartments in the same cell type (vertical axis). Each line represents a TAD clique. **G**: Numbers of TAD cliques belonging to each of the clusters in D.

Next, we analyzed significantly up- and downregulated genes in conjunction with the corresponding subcompartment switches. This shows that at specific chromosomal regions (Fig. 2B), or at genome-wide levels (Fig. 2C–F), there are correlated patterns of subcompartment switching and differential gene regulation. Inspecting the presence of differentially downregulated genes in T1 cells relative to 10A cells, reveals that B1(10A)→B2(T1) is the most frequent switch for these genes (Fig. 2C upper left panel). In addition, switching between B0/A0/A1(10A)→B1(T1) or from A1(10A)→B1/B0/A0(T1) is enriched relative to what is seen for non-differentially expressed genes (Fig. 3C top middle panel) or upregulated genes (Fig 2C top right panel). To quantify whether up- and down-regulated genes tends to involve subcompartment switches towards more open (or A-like) subcompartments, or closed (or B-like), respectively, we computed the log-ratio of subdiagonal sums in the upper-compared to the lower triangular matrices from Fig. 2C. When this log-ratio is negative, DE genes are more present in 10A, and when it is positive, DE genes are more present in T1 (Fig. 2D) or C1 (Fig. 2F). Thus, comparing this ratio for down-regulated, non-differential and up-regulated genes for subcompartment switches of different types allows quantifying whether subcompartment switching is systematically associated with genes that are up- or down-regulated. Indeed, this analysis confirms the trend of switching towards more open subcompartments for upregulated genes (Fig. 2D; left) and vice versa for downregulated genes (Fig. 2D; right). When contrasting 10A and C1, similar trends are seen (Fig. 2E). As expected, no trend is seen for non-differentially expressed genes (Fig. 2D and 2E; middle). In conclusion, subcompartment switching corresponds with changes in gene transcription in the breast cancer progression model.

### Reorganization of TAD-TAD interactions coincides with subcompartment deterioration

Beyond their subcompartment organization, we have previously shown that TADs can arrange in densely connected long-range contact configurations termed TAD cliques (Paulsen et al. 2019). Using a similar approach, we computed TAD cliques in 10A, T1 and C1 cells (see Fig. 3A), and identified a substantial number of TAD cliques in all cell types. The distribution of maximal clique sizes is generally similar across the three cell stages (Fig. 3B; Supplementary Fig. S23).

We overlapped TAD cliques of different sizes with subcompartments, and observed that association of B-like subcompartments increases with TAD clique size, to the extent that large TAD cliques involving >6 TADs almost exclusively consist of TADs in B3 subcompartments in 10A (Fig. 3C). This trend aligns with our previous subcompartment characterization of TAD cliques and reaffirms that TAD cliques are distinct from subcompartments (Liyakat Ali et al. 2021). Comparing subcompartment associations across the three stages, reveals that cliques of size >5 are generally B2-enriched in T1 and C1, and thus the subcompartment composition of larger cliques is altered in these two stages relative to 10A (Supplementary Fig. S24).

To characterize TAD clique dynamics, we analyzed alterations in TAD maximal clique sizes across cell types, visualized as alluvial plots (Fig. 3D–E; Supplementary Fig. S25–S32). This analysis revealed that a large fraction of TADs either become TAD cliques (Fig. 3D), or grow or decrease in TAD clique size (Fig. 3E; Supplementary Fig. S25–S32) during the three stages. Thus, like subcompartments, TAD cliques also reconfigure extensively during breast cancer progression in this model system.

To explore the extent to which TAD cliques bring together TADs with distinct subcompartments, we used HDBSCAN (McInnes et al. 2017; McInnes and Healy 2017) to cluster each clique based on their TAD-wise enrichment in subcompartments. Clustering was performed targeting 5 clusters with a minimum cluster size of 100 TAD cliques, plus a cluster dedicated to collect outliers (see Supplementary Fig. S33). The assigned clusters (see Fig. 3F) show that individual TAD cliques frequently involve multiple types of subcompartments in the same clique. Singleton (non-clique) TADs generally overlap with a single subcompartment only (Supplementary Fig. S34), and thus TAD cliques represent a level of TAD association where subcompartments of different types potentially intermix.

To investigate the dynamics of TAD clique clusters, we explored the frequency of each of the five TAD clique clusters in each of the three cancer stages (Fig. 3G; Supplementary Table S6). This comparison revealed that most clusters display a cancer progression stage-specific association, such that a cluster tends to be more enriched in one or two of the three cell types examined here (Fig. 3G). Specifically, cluster 0, which contains TAD cliques rich in A3, A1 and B0 subcompartments (Fig. 3F), is gradually reduced in T1 and C1 (Fig. 3G). Clusters 2 and 3, which harbor TAD cliques associated with subcompartments A0-A2 (Fig. 3F), are depleted in T1 and C1 (Fig. 3G). Cluster 4 gradually increases in T1 and C1 (Fig. 3F), and is associated with subcompartments B0, B1 and B2 (Fig. 3G).

In summary, TAD cliques bring together TADs overlapping diverse subcompartments. Whereas the number and size of TAD cliques stay generally similar across the cancer stages, they associate differently with subcompartments. Specifically, large B3-enriched TAD cliques in 10A are replaced by B2-enriched cliques in T1 and C1. Simultaneously, TAD cliques bringing together diverse A-subcompartments in 10A are reduced in T1 and C1. Generally, this signifies the role of subcompartment deterioration and intermixing during breast cancer progression.

### Interchromosomal insertion of the *MYC* locus evokes de novo enhancer contacts and oncogene activation

**Fig. 4.**
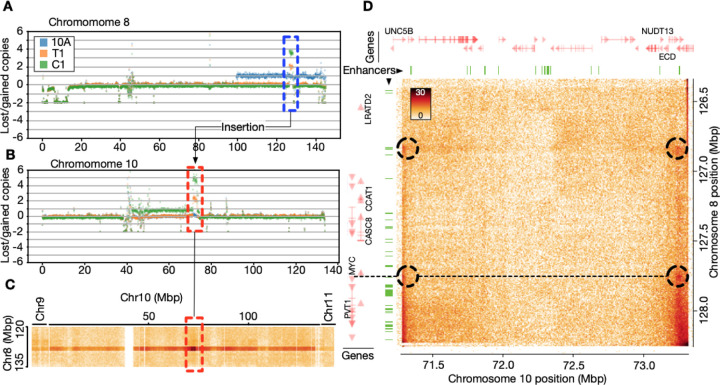
De novo enhancer formation upon *MYC* locus insertion on chromosome 10. **A**: Plot showing lost/gained copies for the entire chromosome 8 in the three cell types. Blue dotted region highlights specific amplification of the chromosome 10 region. **B**: Plot showing lost/gained copies for the entire chromosome 10 in the three cell types. Red dotted region highlights specific amplification of the chromosome 8 region. **C**: Interchromosomal C1 Hi-C contacts between the region on chromosome 8 (vertical axis) and the entire chromosome 8. Highlighted region from B indicated in red. End of chromosome 9 and beginning of chromosome 11 shown on the left and right side, respectively. **D**: Zoom-in on the C1 Hi-C map of the chromosome 8 – 10 amplification unit. Dotted circles indicate enriched “dots” of contacts involving enhancers and *MYC*. Dotted line shows position of *MYC* gene on chromosome 8 relative to contacts on chromosome 10 within the amplification unit. Positions of enhancers from MCF10A indicated as green segments. Genes shown in red, with names for selected genes.

As expected in cancer, the cells in our breast cancer progression model harbors structural variations (SVs), including copy number alterations, insertions, deletions and translocations. We exploited the ability of Hi-C to detect many of these SVs, to call SVs genome wide (see suppl. Fig S35–S37). We manually inspected the called variants and confirmed known SVs (Santner et al. 2001), including t(3;9), t(3;5), t(3;17), t(6;19). In addition, we identified previously undescribed SVs, including t(7;9) and t(10;17). Analyzing the 3D structural consequences of all SVs in these cell lines is computationally infeasible. Nevertheless, to investigate 3D genome consequences of select SVs, we focused on a striking pair of regions on chromosome 8 (126330000-128235000 bp; hg38) (Fig. 4A) and chromosome 10 (71280000-73310000 bp; hg38) (Fig. 4B). Both regions show a gradual and coordinated increase in copy numbers going from T1 to C1 (Fig. 4A,B). The region on chromosome 8 is well-characterized and contains several breast cancer related genes, including *MYC*, an oncogenic transcription factor playing a pivotal role in breast cancer progression (Liao and Dickson 2000) and whose amplification and overexpression is a marker of aggressive and invasive breast cancer (Corzo et al. 2006; Berns et al. 1992).

Upon further inspection of the Hi-C data at the intersection of these two regions, we noticed general enrichment of contacts of the region towards the entire chromosome 10 after ICE balancing, clearly indicating that the *MYC* locus is inserted in the chromosome 10 region (Fig. 4C). We zoomed in on the interchromosomal contacts and noticed patterns resembling dots and stripes typically seen for looping and extruding intra-chromosomal DNA, again indicating an insertion event (Fig. 4D). By inspecting subcompartments called for this region, the insertion site on chromosome 10 is found to be entirely covered by A3-subcompartments (Supplementary Fig. S38A) in all three cell types, indicating that the *MYC* locus is inserted into a highly active genome region. Inspecting subcompartments surrounding MYC reveals a switch from A2 to A3 subcompartments from 10A to T1 and C1 (Supplementary Fig. S38B).

Overlaying positions of MCF10A enhancers from EnhancerAtlas 2.0 (Gao and Qian 2020) onto the chromosome 8 and 10 amplification unit, the four dots seen in the Hi-C map evidently corresponds to two *de novo* contact points between enhancer elements on chromosome 10 and the promoter of *MYC*, and an interaction with enhancer elements ~1 Mbp upstream of the *MYC* promoter (Fig. 4D). Inspecting RNA-seq data for these two regions shows an upregulation of breast cancer related genes in T1 and C1 relative to 10A, including *LRATD2, PCAT1, CASC19, CASC8, POU5F1B, PVT1* and *MYC* on chromosome 8. A range of breast cancer related genes in the chromosome 10 region are also upregulated, including *UNC5B, CDH23, PSAP, SPOCK2, ASCC1, DDIT4, NUDT13, ECD* and others (see supplementary Tables S7 and S8).

In conclusion, our results highlight how structural variants in the genome can work together with 3D genome states to drive oncogenesis.

## Discussion

The intricate 3D organization of the genome plays a pivotal role in cellular function and gene regulation, and therefore not surprisingly, is receiving more attention in the context of cancer research. To date, no study has examined the 3D chromatin organizational changes associated with the progression of breast cancer. Here, we addressed this knowledge gap using a multilayered genomic and epigenomic approach and uncovered four major aspects to the disruption of the higher-order structure of chromatin that occurs when a normal human breast epithelial cell line is transformed into a malignant state.

First, major compartment and subcompartment switching emerges as a prominent dynamic higher-order genomic feature during breast cancer progression. This subcompartment switching is correlated with altered expression of genes implicated in breast cancer thereby potentially driving the disease’s progression.

Second, we identify a reconfiguration of TAD cliques during breast cancer progression, and these changes bring together new subcompartment-associations involving both B- and A-subcompartments.

Third, we show that spatial sub-compartment 3D nuclear organization degenerates in the pre-malignant and malignant states likely also contributing to the loss of gene expression control. Collectively, there is a coordinated and progressive reorganization of higher-order genomic structure to an abnormal state.

Finally, we reveal a previously unknown translocation event where a locus on chromosome 8 containing the *MYC* oncogene is inserted into highly active A3-subcompartments on chromosome 10. This results in *de novo* enhancer-promoter interactions correlating with not only elevated *MYC* expression in T1 and C1, but also enhanced expression of other breast cancer related genes on both chromosomes. Note, the mirror-symmetry of contacts on the chromosome 10 segment with specific regions on the chromosome 8 segment (Fig. 4D), could also suggest that the whole region exists as circular, extrachromosomal DNA (ecDNA), which is a common form of oncogenic amplification, and known to often occur around *MYC* (Hung et al. 2022; Hoff et al. 1988). The fact that the region exists as an almost isolated contact map in the interchromosomal Hi-C data further supports this hypothesis. Interestingly, ecDNA has been shown to promote functional cis-regulatory contacts (Morton et al. 2019), or in hubs (Hung et al. 2021), to drive oncogenesis.

Beyond our identified interchromosomal insertion of the *MYC* locus, the intricate aneuploidy patterns commonly observed in cancer pose a challenge that existing standard software and pipelines struggle to effectively address. Promising strides have been made using long-read Hi-C technology to address this issue (Garg 2023). Nevertheless, a significant gap remains in the availability of computational pipelines tailored for comprehensive downstream analysis of reordered cancer genome data relative to a karyotypically normal reference genome. This needs to be addressed if further progress in understanding the 3D cancer genome is to be achieved.

In conclusion, our findings provide new insights into the complex genomic structural changes that underlie breast cancer metastatic progression. The interplay between subcompartment switching, LAD-linked subcompartment spatial nuclear reorganization, and TAD clique dynamics represents a multifaceted mechanism by which the 3D genome can be abnormally and dynamically reconfigured during breast cancer development. Targeting these 3D genome alterations could potentially lead to new therapeutic strategies to better treat breast cancer in the future (Park et al. 2023).

## Methods

### Cells

MCF10A, MCF10AT1, and MCF10Ca1a cell lines were grown in DMEM/Nutrient F12 (DMEM/F12) media supplemented with 5% horse serum (MCF10A and MCF10Ca1a) or 2.5% horse serum (MCF10AT1), 14 mM NaHCO3, 10 μg/mL insulin, 2 mM L-glutamine, 20 ng/mL human epidermal growth factor, 500 ng/mL Hydrocortisone and 100 ng/mL cholera Toxin. MCF10A cells were obtained from the American Type Culture Collection (CRL-10317). MCF10AT1 and MCF10Ca1a cells were obtained from the Barbara Ann Karmanos Cancer Institute (Detroit, Michigan).

### Hi-C

HiC Libraries were prepared in duplicates for each cell type using the Arima-HiC+ kit (Arima Genomics, USA) according to manufacturer instructions. For each cell line (10A, T1, C1) 1 million cells were used per HiC reaction. Cells were fixed with 1.2% formaldehyde for 12 min. All subsequent steps were carried out according to the Arima protocol. Resulting libraries were amplified with 5 PCR cycles and sequenced on an Illumina NovaSeq 6000 instrument in paired-end run with 2 ×101 bp.

### RNA-seq

All mRNA-Seq experiments were performed in triplicate. Total RNA was isolated using the Qiagen RNeasy kit following manufacturer’s instructions. Stranded mRNAseq libraries were constructed using Illumina mRNA Prep kit, following vendor protocol with poly-A enrichment (Illumina). Libraries were sequenced with 2×75bp paired-end on Illumina NextSeq 500 instrument.

### ChIP-seq of lamin B1 and mapping of lamina-associated domains (LADs)

ChIP of lamin B1 was done as described by us (Rønningen et al. 2015). In short, cells were cross-linked with 1% formaldehyde, lysed in 50 mM Tris-HCl pH 7.5, 10 mM EDTA, 1% SDS and protease inhibitors, and sonicated in a Bioruptor (Diagenode) into ~200-bp fragments. After sedimentation, the supernatant was diluted tenfold in RIPA buffer. Chromatin was incubated overnight at 4°C with antibodies to lamin B1 (10 μg per 10 million cells; Abcam, ab16048), coupled to Invitrogen Dynabeads protein A/G (Thermo-Fisher). ChIP samples were washed four times in ice-cold RIPA buffer, after which cross-links were reversed and the DNA was eluted for 6 h at 68°C. DNA was purified using phenol–chloroform isoamylalcohol and dissolved in H2O. ChIP-seq libraries were prepared using the Diagenode Microplex library preparation kit v2 and TruSeq LT indexes, and samples were sequenced (single-end) on an Illumina HiSeq4000.

ChIP sequence reads were mapped to hg38 with Bowtie2 v2.4.1 (https://github.com/BenLangmead/bowtie2) after removing duplicates using Picard MarkDuplicates (http://broadinstitute.github.io/picard/). Reads from both input samples were merged, mapped to HG39 and duplicates removed as above. To alleviate normalization bias, each pair of mapped ChIP and input read files contained the same read depth after down-sampling reads for each chromosome. Mapped reads were used to call LADs from ten consecutive runs of Enriched Domain Detector (EDD) (http://github.com/CollasLab/edd) (Lund et al. 2014) with auto-estimation of GapPenalty and BinSize, and mean GapPenalty and BinSize values were used for a final EDD run (Forsberg et al. 2019). Final LADs for each cell type were the merge of the three replicates.

### Structure of code used for data analyses

Source code used to produce all results presented in this paper (except LADs analysis and downstream analysis of Chrom3D models) is hosted on GitHub at github.com/paulsengroup/2022-mcf10a-cancer-progression and is archived on Zenodo at doi.org/10.5281/zenodo.10069549.

Most of the computation is organized into Nextflow workflows, with each workflow performing a subset of the data analysis (e.g. the differential expression and comparative analyses are performed by two different workflows).

All workflows mentioned in the method sections are hosted on the GitHub repository inside folder workflows/. Scripts and Jupyter notebooks are found under folders bin/ and notebooks/ respectively.

Some of the steps required us patching third party tools. The patches are described in section Software patches and patch files are available on the GitHub repository under container/patches/. All our patches were contributed upstream. Most of our patches were accepted by upstream and are already part of a stable release. The draft version of most figures were produced directly by workflows or scripts under bin/plotting. The final version of figures were produced by manually assembling draft images with Inkscape and Omnigraffle.

### Hi-C preprocessing

We used a modified version of nf-core/hic v2.0.0 (https://zenodo.org/records/2669513) (Ewels et al. 2020; Servant et al. 2015; Ewels et al. 2016; da Veiga Leprevost et al. 2017) for quality control, sequence mapping and filtering using hg38.p14 as reference genome (Lander et al. 2001). The workflow was run using restriction enzymes for the 2-enzyme Arima Kit (--restriction_site=“ĜATC,GÂNTC” and --ligation_site=“GATCGATC,GANTGATC,GANTANTC,GATCANTC”). The following optional flags were specified: --skip_maps, --skip_dist_decay, --skip_tads, --skip_compartments, --skip_balancing, --skip_mcool, --split_fastq=false. The .chrom.sizes file given to nf-core/hic was filtered to only retain chromosome sequences using grep ‘ĉhr[[:digit:]XY]\+[[:space:]]’. Chromosomes were sorted by name using gnu sort -V. The output of nf-core/hic was compressed using workflow robomics/compress-nfcore-hic-output v0.0.1 (https://zenodo.org/records/7949266). Hi-C contact matrices in multi-resolution Cooler format were generated using a patched version of cooler v0.9.1 (Abdennur and Mirny 2020). We used cooler cload to ingest interactions in .validPair format into a .cool file at 1 kbp resolution. We then converted the .cool file to .mcool file format using cooler zoomify. Finally, matrices were balanced with several methods using juicer_tools v2.20.00 (Durand et al. 2016) (VC, KR, SCALE methods using intra-chromosomal, inter-chromosomal and genome-wide interactions), and cooler (ICE method using intra-chromosomal, inter-chromosomal and genome-wide interactions) (Abdennur and Mirny 2020). Regions overlapping centromeres and assembly gaps for hg38 were masked before balancing (files retrieved from UCSC FTP server on 2023/02/24). Downstream analyses were performed using cis-only, ICE balanced matrices unless otherwise specified. For some analyses, replicates for the same condition were merged to generate deeper Hi-C matrices using cooler merge (Abdennur and Mirny 2020). The resulting matrices were then coarsened and balanced cooler and juicer_tools as outlined above (Abdennur and Mirny 2020; Durand et al. 2016). All the above steps were performed by running script run_nfcore_hic.sh, which runs workflow postprocess_nfcore_hic.nf after running nf-core/hic. Fig. S1 was generated by workflow postprocess_nfcore_hic.nf based on the output of nf-core/hic. Fig. S2 was generated by workflow comparative_analysis_hic.nf by running the Python implementation of hicrep v0.2.6 (Lin et al. 2021; Yang et al. 2017). Correlation values shown in the plot were computed as the weighted average of the correlation coefficient of each individual chromosome using chromosome sizes as weights.

### RNA-seq preprocessing

We used nf-core/rnaseq v3.12.0 (https://zenodo.org/records/7998767) to perform quality control, trimming, alignment and generating the gene expression matrix (Ewels et al. 2020; Di Tommaso et al. 2017; Quinlan and Hall 2010; Chen et al. 2018; Liao et al. 2014; Pertea and Pertea 2020; Ewels et al. 2016; Daley and Smith 2013; Okonechnikov et al. 2016; Li and Dewey 2011; Wang et al. 2012; Patro et al. 2017; Li et al. 2009; Kopylova et al. 2012; Dobin et al. 2013; Kovaka et al. 2019; Kent et al. 2010; Love et al. 2014; Sayols et al. 2016; Love et al. 2020; da Veiga Leprevost et al. 2017; Kurtzer et al. 2017). nf-core/rnaseq was run using hg38.p14 as reference genome (Lander et al. 2001). The FASTA file used as reference was first filtered using SeqKit v2.5.1 (Shen et al. 2016) to remove mitochondrial chromosomes and unplaced scaffolds by using the following pattern ‘ĉhr[XY\d]+$’. We used the genome annotation for hg38 from Gencode v43 (Frankish et al. 2019). The pipeline was launched using the following optional flags: --aligner=star_salmon, --pseudo_aligner=salmon, --extra_salmon_quant_args=‘--seqBias --gcBias’, --gencode=true. The workflow was run by launching script run_nfcore_rnaseq.sh.

### ChIP-seq analyses of public datasets

We used nf-core/chipseq v2.0.0 to perform quality control, mapping, filtering and peak calling of publicly available ChIP-seq datasets (Di Tommaso Paolo Nahnsen Sven; Ewels et al. 2020; Di Tommaso et al. 2017; Li and Durbin 2009; Barnett et al. 2011; Ramírez et al. 2016; Liao et al. 2014; Heinz et al. 2010; Zhang et al. 2008; Ewels et al. 2016; Landt et al. 2012; Daley and Smith 2013; Li et al. 2009; Kent et al. 2010; Love et al. 2014; Wickham 2007; da Veiga Leprevost et al. 2017; Kurtzer et al. 2017). The pipeline was run using hg38.p14 as reference genome from UCSC (Lander et al. 2001) and the gene annotation from Gencode v43 (Frankish et al. 2019). BWA was used for mapping and effective genome size for MACS2 was computed using unique-kmers.py from khmer v2.1.1 (Li and Durbin 2009; Zhang et al. 2008; Crusoe et al. 2015; Döring et al. 2008). Option --narrow_peak was specified when processing ChIP-seq datasets for CTCF and p53.

The workflow was launched by executing script run_nfcore_chip.sh.

### Calling of copy number variations

Copy number variations (CNVs) were called from Hi-C data using HiNT-CNV v2.2.8 (Wang et al. 2020).

The list of restriction sites for the Arima 2-enzyme kit was generated by running script bin/compute_restriction_sites_for_hint.py on hg38.14 (Lander et al. 2001).

Reference data and background matrices used by HiNT were downloaded from compbio.med.harvard.edu/hint.

Data analysis steps are defined in workflow detect_structural_variants which was run by launching script run_detect_structural_variants.sh.

### Annotation of structural variations

Large structural variations such as translocations were manually annotated by us by looking for square regions of enriched interactions and sharp transitions in the trans portion of the Hi-C matrix (see Supplementary Tables S9–11).

### TAD analyses

We determined genomic positions of topologically associated domains (TADs) in all samples with HiCExplorer v3.7.2 using default parameters on matrices at 10, 20, 50 and 100 kbp resolutions (Ramírez et al. 2018; Wolff et al. 2018, 2020). Workflow tad_analysis.nf was used to generate the draft figures that went into supplementary figures S2–6. The same workflow was also used to run HiCExplorer. Draft figures for Fig. 1A was generated using notebook bin/plotting/plot_tads_higlass.ipynb. Supplementary Fig. S5 was generated by fetching interactions at 50 kbp resolution for each 10A TAD across cell types, masking values overlapping the first 150 kbp around the diagonal. Finally interactions were summed and normalized by TAD size (as in, the number of pixels overlapping a TAD). To generate Supplementary Fig. S6, TADs from two cell types were paired based on the highest overlap (Jaccard Index of genomic coordinates), and the distribution of pairwise overlaps were plotted. TADs overlapping or around regions masked by matrix balancing were not considered, as the insulation score can be unreliable in these regions.

The analysis is encapsulated by workflow tad_analysis.nf which was run by launching script run_tad_analysis.sh.

### TAD clique analyses

The TAD annotation generated by HiCExplorer was given as input to workflow github.com/robomics/call_tad_cliques v0.3.1 (https://zenodo.org/records/8308245) to identify cliques of TADs as outlined in Paulsen et al. (Paulsen et al. 2019). TAD cliques were called across all three cell types using TADs from 10A. This is a necessary simplification required to allow comparing cliques across cell types in downstream analyses. The output of github.com/robomics/call_tad_cliques was used as input for workflow postprocess_call_tad_cliques.nf, which generated the draft version of Fig. 3B, 3D, 3E and supplementary figures S23,S25–32.

Workflows were launched by running script run_call_tad_cliques_workflow.sh.

### Subcompartment analyses

Subcompartments were determined using a patched version of dcHiC d4eb244 (Chakraborty et al. 2022). A and B compartment annotation was generated from subcompartments by aggregating A-subcompartments and B-subcompartments. The dcHiC processing pipeline was wrapped in workflow compartment_analysis.nf, which converts matrices in .mcool file to a format understood by dcHiC and runs all analysis steps except those for --pcatype=trans and --pcatype=enrich. When appropriate, the reference genome assembly, annotation and .chrom.sizes for hg38.p14 were provided to dcHiC through the --gfolder option. We fixed the seed used by dcHiC to ensure our results are reproducible by others. Subcompartments were called at the following resolutions: 10 kbp, 20 kbp, 50 kbp, 100 kbp, 200 kbp and 500kbp. However, only subcompartments at 10 kbp resolution were used for further data analyses. The same workflow was used to generate the draft figures for Fig. 1B and 1D as well as supplementary figures S9–11. Fig. 1C was generated using the output of the viz step of dcHiC as the starting point.

Figure S9–11 were generated by tracking subcompartment assignments across cell types. Bins were paired based on their genomic coordinates, then subcompartment switches were classified into 5 different classes: neutral (i.e. no switch), reverted (e.g. switch occurred in T1 but was reverted in C1), transition to open/close and other switches (e.g. partial reversion: A3->A1->A2). Subcompartment switches were further classified using a delta score ∆_*ij*_ defined as follows. We quantify the degree of switching between two stages, i and j (∆_*ij*_), by assigning ranks to the subcompartments (B3=0…A3=7) and calculating the rank difference. A negative rank difference (∆_*ij*_) thus indicates a transition towards B-like subcompartments, indicating chromatin closure. Conversely, a positive value signifies a shift towards A-like compartments, indicating chromatin opening.

Data analysis steps are defined in workflow compartment_analysis which was run by executing script run_compartment_analysis.sh.

### Differential expression analyses

Differential expression analysis was performed using DESeq2 v1.38.0 and apeglm v1.20.0 (Love et al. 2014; Zhu et al. 2019).

DESeq2 was called with default parameters using the raw count table produced by nf-core/rnaseq as input. Log2-fold-change shrinkage estimation was computed with the lfcShrink function from DESeq2 using apeglm as shrinkage estimator and the following log2FoldChange cutoffs: 0.0, 0.1, 0.25, 0.5, 1.0, 1.5, 2.0, 2.5, 3.0, 3.5, 4.0, 4.5, 5.0.

The analysis was automated using workflow diff_expression_analysis.nf, which was launched with run_diff_expression_analysis.sh.

Supplementary figure S21 was generated by running script run_cluster_profiler_do.py. The script uses clusterProfiler v4.8.1 (Wu et al. 2021; Yu et al. 2012) and the disease ontology (DO) database from the DOSE v3.26.1 package (Yu et al. 2015) to perform over-representation analysis of DO terms (Schriml et al. 2023). Package enrichplot v1.20.0 ([CSL STYLE ERROR: reference with no printed form.]) was used for visualization. The IDs of differentially expressed genes were provided as input to clusterProfiler. Genes were considered as differentially expressed based if they exhibit an absolute log2FoldChange value of 0.5 or greater and a p-value of 0.01 or smaller. Furthermore, clusterProfiler was run using a q-value of 0.05.

### Comparative analyses

All comparative analyses were performed using workflow comparative_analysis.nf.

Fig. S8 was generated by overlapping subcompartment labels with several different epigenetic markers. First, each ChIP-seq peak was assigned a score by summing the raw ChIP-seq signal over each peak, then each peak was assigned to a subcompartment. Finally peaks were grouped by subcompartment label and the mean signal was computed for each subcompartment.

Fig. 2A and supplementary Fig. S22 were generated by overlapping expression levels in TPMs with subcompartment labels overlapping genes from Gencode v43 for hg38. In case a gene was tagged with multiple subcompartment labels, the gene was assigned the label with the largest coverage. In case of coverage tie, the gene was discarded (note that this is an extremely rare occurrence).

Figure 2B was generated using pyGenomeTracks (Ramírez et al. 2018; Lopez-Delisle et al. 2021) to visualize up/down regulated genes overlapping a region involved in subcompartment switching.

Figures 2C–F were generated by overlapping subcompartments with differentially expressed genes across all three cell types. First genes were assigned a subcompartment label by overlapping their TSS with the subcompartment annotation generated by dcHiC. Next genes were grouped based on log2FoldChange and p-value in down-regulated, non-differentially expressed and up-regulated genes (lfc=2.0; pvalue=0.01). Finally each group of genes was plotted as heatmaps showing subcompartment switches across pairs of cell types (genes not involved in subcompartment switches are not shown). Figures 2D and 2F were generated from the table underlying heatmaps 2C and 2E as follows: compute the sum of genes for each diagonal *i*; starting from diagonal *i* = 1, pair diagonal i with diagonal − *i*; finally compute the log2 ratio between the sum of diagonal *i* and the sum of diagonal − *i*. Positive values indicate a positive correlation between one of the classes of differentially expressed genes and switches towards open chromatin while negative values indicate a positive correlation with subcompartment switches towards close chromatin.

The draft figure for Fig. 3A was generated with bin/plotting/plot_tad_cliques.py using TAD cliques, TADs, compartment PCA and gencode v43 gene annotation as input.

The draft figures for Fig. 3C and supplementary Fig. S24 were generated by overlapping subcompartment states with TADs annotated with the size of the largest clique to which they belonged to.

The draft figures for Fig. 3F–G and supplementary Fig. S33–34 were generated by clustering TADs and TAD cliques using HDBSCAN (McInnes et al. 2017; McInnes and Healy 2017) based on their subcompartment composition.

First, domains (that is TADs or TAD cliques) were annotated with their subcompartment state composition by overlapping subcompartment states with the domains using annotate_domains_with_subcompartments.py. This resulted in a count matrix with one row per domain, where each row counts the number of bins labeled for each subcompartment state. For example, given a domain overlapping 3 A1 bins, 10 A2 bins and 2 A3 bins, the entry in the count matrix corresponding to this domain would be 0, 0, 0, 0, 0, 3, 10, 2.

Next, this count matrix was given as input to cluster_domains_by_subcompartment_state.py, which clustered domains using HDBSCAN based on their similarity in subcompartment composition. Clustering was performed targeting 5 clusters with a minimum cluster size of 100 domains, plus a cluster dedicated to collect outliers. Finally, clusters were visualized using plot_domain_subcompartment_clusters.py.

All comparative analyses are defined in workflow comparative_analysis.nf which was launched with script run_comparative_analysis.sh.

### 3D genome modeling

A patched version of Chrom3D v1.0.2 was used to generate 3D genome models for 10A, T1 and C1 (Paulsen et al. 2017). Centromeres, assembly gaps, and translocations were filtered from the TAD domains that were subsequently utilized for Chrom3D simulations. A list of interacting TAD domains was created using interacting domain pairs in TAD cliques, which was used in Chrom3D simulations as spatially proximal interaction constraints. LADs were likewise matched to the corresponding TAD domains and used as peripheral sub-nuclear constraints in the simulations. Significantly interacting TADs and LADs were converted to Gtrack files using the Python scripts makeGtrack.py and make_diploid_gtrack.py, which were used as Chrom3D inputs. A total of 100 Chrom3D simulations were run for each condition with a nuclear occupancy of 0.15, at TAD resolution and 2 million simulation (Paulsen et al. 2017) steps. A unique seed number was generated using the script generate_seed_sequence.py for each Chrom3D simulation. In addition, the optional parameter --nucleus was set to push the beads towards the nuclear interior. The analysis was automated through the workflows robomics/call_tad_cliques and run_chrom3d.nf, which were launched with the script run_chrom3d.sh.

ChimeraX was used to visualize the simulated Chrom3D models (Pettersen et al. 2021). The median distance of each chromosome from the nuclear center was calculated for each Chrom3D simulation and visualized using ggplot2 (Villanueva and Chen 2019). The median distance and the standard deviation of each sub-compartment were likewise evaluated for each Chrom3D simulation and visualized using ggplot2. Sub-compartment distances from the nuclear center were statistically evaluated and compared within conditions using the Wilcoxon rank sum test in R with the function wilcox.test.

### Software used throughout data analysis code

The following software packages were used throughout the code used for data analysis:

- bedtools (Quinlan and Hall 2010) - to perform common set operations on genomic intervals.
- bedGraphToBigWig (Kent et al. 2010) - to convert bedgraph files to bigWig format.
- bioframe (Open2C et al. 2022) - to perform common set operations on genomic intervals.
- matplotlib (Hunter May-June 2007) - to generate plots.
- NumPy (Harris et al. 2020) - to efficiently perform arithmetic operations on vector data.
- pandas (McKinney 2010) - to read, write and manipulate tabular data using dataframes.
- pyBigWig - to read and write bigWig files.
- Scipy (Virtanen et al. 2020)- to compute Pearson correlation values.
- samtools (Danecek et al. 2021) - to index FASTA files and perform IO operations on SAM, BAM and CRAM files.
- Seaborn (Waskom 2021) - to generate plots.

### Software patches

This section describes the purpose of the patches we applied to nf-core/hic, dcHiC and cooler. Patch files are available under containers/patches.

Patch for nf-core/hic v2.0.0 (https://zenodo.org/records/2669513)

Our patch involved updating the pipeline to correctly handle the --restriction_site and --ligation_site parameters.

Our patch was accepted by upstream and is now part of v2.1.0 of the pipeline. See nf-core/hic/pull/153 for more details.

Patches for dcHiC d4eb244 (Chakraborty et al. 2022)

We patched dcHiC as follows:

- Define a default seed and introduce a --seed CLI option to ensure results are reproducible across runs
- Wrap calls to depmixS4::fit into a try-catch block to attempt model fitting up to 5 times in case of spurious failures
- Properly handle the possibility that a chromosome does not have any bin overlapping one or more subcompartment type.

Our patches were accepted by upstream but are not yet part of a stable release. See ay-lab/dcHiC/pull/59, ay-lab/dcHiC/pull/60, ay-lab/dcHiC/pull/62 and containers/patches/dchic.patch for more details.

Patch for cooler v0.9.1 (Abdennur and Mirny 2020)

We patched cooler to ensure that convergence of cooler balance using cis-only interactions was correctly reported for all chromosomes instead of just the last one.

When balancing interactions using the --cis-only flag, cooler balances interactions for each chromosome independently. Given that there is no guarantee that ICE will converge within the given number of iterations, cooler balance stores an attribute inside balanced cooler files to report whether balancing was successful (i.e. convergence was achieved).

In cooler v0.9.1 there is a bug in the logic that writes this attribute that causes the convergence for the last chromosome balanced to be reported instead of the convergence status for each chromosome. Our patch addresses this bug such that the convergence status of matrices balanced with --cis-only can be assessed correctly.

The patch also addressed some minor issues related to detecting pandas’ version at runtime and handling of bin sizes represented using unsigned integers.

Our patches were accepted by upstream and are now part of cooler v0.9.2.

See open2c/cooler/pull/313, open2c/cooler/pull/323, open2c/cooler/pull/324 and containers/patches/cooler.patch for more details.

Patch for Chrom3D v1.0.2 (Paulsen et al. 2017)

We patched Chrom3D to support building the project using CMake and addressing a few compiler warnings and errors that were preventing us from compiling Chrom3D with a modern C++ compiler toolchain. See containers/patches/chrom3d.patch for more details.

Patch for HiNT v2.2.8 (Wang et al. 2020)

We patched HiNT to support processing Hi-C matrices using Arima restriction enzymes. See containers/patches/hint.patch for more details.

### Data Access

All raw and processed sequencing data generated in this study have been submitted to the NCBI Gene Expression Omnibus (GEO; https://www.ncbi.nlm.nih.gov/geo/). Unique persistent identifier and hyperlink to datasets are listed below:

- RNA-seq:
  - To review GEO accession GSE246947:
  - Go to https://www.ncbi.nlm.nih.gov/geo/query/acc.cgi?acc=GSE246947
  - Enter token xxx into the box
- ChIP-seq of Lamin1 (LADs):
  - To review GEO accession GSE246599:
  - Go to https://www.ncbi.nlm.nih.gov/geo/query/acc.cgi?acc=GSE246599
  - Enter token xxx into the box
- ChIP-seq (reanalysis) of histone modification data:
  - To review GEO accession GSE247171:
  - Go to https://www.ncbi.nlm.nih.gov/geo/query/acc.cgi?acc=GSE247171
  - Enter token xxx into the box
- Hi-C:
  - To review GEO accession GSE246947:
  - Go to https://www.ncbi.nlm.nih.gov/geo/query/acc.cgi?acc=GSE246947
  - Enter token xxx into the box

## Supplementary Material

Supplement 1

Supplement 2
