# Supplementary material for: Loss of multi-level 3D genome organization during breast cancer progression
Source: bioRxiv. 2024 Aug 8:2023.11.26.568711. Originally published 2023 Nov 27. Preprint. [Version 2] doi: 10.1101/2023.11.26.568711 (PMC10705249; doi:10.1101/2023.11.26.568711)
Supplement: Supplement 2 [file media-2.pdf]

Loss of multi-level 3D genome organization during breast cancer progression

Supplementary tables

Table S1: Hi-C interactions classified by type.

	MCF10A_R1	MCF10A_R2	MCF10AT1_R1	MCF10AT1_R2	MCF10ACA1a_R1	MCF10ACA1a_R2
reported_pairs	694.20	815.52	837.73	718.09	672.54	786.82
valid_interaction	664.17	774.48	821.28	704.73	650.15	760.91
valid_interaction_rmdup	598.02	681.51	687.67	603.61	559.65	639.96
trans_interaction	95.93	109.50	112.73	101.96	135.70	158.56
cis_interaction	502.10	572.01	574.94	501.65	423.95	481.40
cis_shortRange	106.91	121.64	105.22	89.85	75.64	85.27
cis_longRange	395.19	450.37	469.72	411.80	348.31	396.13
cis_trans_ratio	5.23	5.22	5.10	4.92	3.12	3.04
cis_long_short_ratio	3.70	3.70	4.46	4.58	4.61	4.65
pct_valid_interaction_ff	24.90	24.90	24.93	24.93	24.93	24.93
pct_valid_interaction_rr	24.92	24.92	24.95	24.96	24.96	24.96
pct_valid_interaction_rf	24.77	24.77	24.83	24.85	24.88	24.88
pct_valid_interaction_fr	25.40	25.41	25.30	25.26	25.24	25.24

Table S2: Coverage of compartment switches.

MCF10A	MCF10AT1	MCF10CA1a	Coverage (Mbp)	Rel. coverage (%)
B	B	B	1024.22	37.58%
B	B	A	119.23	4.37%
B	A	B	76.1	2.79%
B	A	A	146.15	5.36%
A	B	B	101.57	3.73%
A	B	A	60.85	2.23%
A	A	B	97.91	3.59%
A	A	A	1099.61	40.34%

Table S3: Genome fraction (Mbp and relative) for WT, T1 and C1 for each of the 8 subcompartments.

Subcompartment	Coverage (Mbp)			Coverage (relative)		
	MCF10A	MCF10AT1	MCF10CA1a	MCF10A	MCF10AT1	MCF10CA1a
B3	164.82	180.49	172.04	5.34%	5.84%	5.57%
B2	509.71	449.93	468.55	16.50%	14.57%	15.17%
B1	517.25	581.01	580.49	16.75%	18.81%	18.80%
B0	355.62	268.17	251.65	11.52%	8.68%	8.15%
A0	213.53	253.13	216.38	6.91%	8.20%	7.01%
A1	546.21	538.50	482.78	17.69%	17.44%	15.63%
A2	398.25	438.56	523.43	12.90%	14.20%	16.95%
A3	382.88	378.47	392.95	12.40%	12.26%	12.72%

Table S4: Wilcoxon rank sum test results of the median subcompartment distance from the nucleus center in each Chrom3D simulation model per condition and subcompartment pair.

Condition 1	Condition 2	Subcomp.	P-value
10A	C1	A3	< 2.2e-16
10A	C1	A2	0.4977
10A	C1	A1	< 2.2e-16
10A	C1	A0	3.175e-12
10A	C1	B0	7.824e-05
10A	C1	B1	4.367e-16
10A	C1	B2	1.982e-12
10A	C1	B3	1.369e-08
10A	T1	A3	< 2.2e-16
10A	T1	A2	8.995e-07
10A	T1	A1	< 2.2e-16
10A	T1	A0	0.001466
10A	T1	B0	1.548e-06
10A	T1	B1	0.0001672
10A	T1	B2	0.6259
10A	T1	B3	3.16e-13
C1	T1	A3	0.0005997
C1	T1	A2	1.586e-07
C1	T1	A1	0.9251
C1	T1	A0	6.97e-06
C1	T1	B0	0.1222
C1	T1	B1	2.925e-10
C1	T1	B2	4.28e-16
C1	T1	B3	0.05464

Table S5: Number of differentially expressed genes (lfc> 0.5; pvalue<0.01).

contrast	condition	genes	DE genes	downreg	upreg
10A	T1	21082	3180	1659	1521
T1	C1	21082	7510	3739	3771
10A	C1	21082	8362	4169	4193

Table S6: Table of subcompartment switches when comparing 10A with T1. Statistical significance was computed using the one-sided binomial test.

Contrast (10A)	Condition (T1)	pvalue (downreg)	pvalue (upreg)
B3	B3	1.000	1.000
B3	B2	0.938	0.500
B3	B1	0.875	0.125
B3	B0	1.000	1.000
B3	A0	1.000	1.000
B3	A1	1.000	1.000
B3	A2	1.000	1.000
B3	A3	1.000	1.000
B2	B3	0.227	0.813
B2	B2	1.000	1.000
B2	B1	1.000	0.059
B2	B0	1.000	0.500
B2	A0	1.000	0.688

B2	A1	1.000	0.500
B2	A2	1.000	1.000
B2	A3	1.000	0.500
B1	B3	0.500	1.000
B1	B2	0.000	0.982
B1	B1	1.000	1.000
B1	B0	1.000	0.046
B1	A0	1.000	0.072
B1	A1	0.945	0.004
B1	A2	1.000	0.125
B1	A3	0.063	1.000
B0	B3	1.000	1.000
B0	B2	0.125	1.000
B0	B1	0.000	0.989
B0	B0	1.000	1.000
B0	A0	0.125	1.000
B0	A1	0.989	0.095
B0	A2	1.000	0.063
B0	A3	0.500	0.500

A0	B3	0.250	1.000
A0	B2	1.000	0.688
A0	B1	0.004	0.975
A0	B0	1.000	0.250
A0	A0	1.000	1.000
A0	A1	0.846	0.166
A0	A2	0.344	0.313
A0	A3	1.000	1.000
A1	B3	1.000	1.000
A1	B2	0.250	1.000
A1	B1	0.172	1.000
A1	B0	0.038	0.961
A1	A0	0.271	0.928
A1	A1	1.000	1.000
A1	A2	0.081	0.500
A1	A3	0.773	0.019
A2	B3	1.000	1.000
A2	B2	1.000	1.000
A2	B1	0.500	1.000

A2	B0	0.500	1.000
A2	A0	0.891	0.938
A2	A1	0.960	0.622
A2	A2	1.000	1.000
A2	A3	0.953	0.868
A3	B3	1.000	1.000
A3	B2	1.000	1.000
A3	B1	1.000	1.000
A3	B0	0.875	0.875
A3	A0	1.000	1.000
A3	A1	0.500	0.997
A3	A2	0.105	0.229
A3	A3	1.000	1.000

Table S7: Table of subcompartment switches when comparing 10A with C1. Statistical significance was computed using the one-sided binomial test.

Contrast (10A)	Condition (C1)	pvalue (downreg)	pvalue (upreg)

B3	B3	1.000	1.000
B3	B2	0.377	0.212
B3	B1	0.969	0.031
B3	B0	1.000	1.000
B3	A0	1.000	1.000
B3	A1	1.000	1.000
B3	A2	1.000	1.000
B3	A3	1.000	1.000
B2	B3	0.828	0.910
B2	B2	1.000	1.000
B2	B1	1.000	0.229
B2	B0	0.637	0.500
B2	A0	0.997	0.623
B2	A1	0.813	0.194
B2	A2	1.000	0.500
B2	A3	1.000	1.000
B1	B3	0.188	1.000
B1	B2	0.000	0.868
B1	B1	1.000	1.000

B1	B0	0.885	0.077
B1	A0	0.997	0.908
B1	A1	0.708	0.001
B1	A2	0.746	0.013
B1	A3	0.969	1.000
B0	B3	1.000	1.000
B0	B2	0.637	1.000
B0	B1	0.196	0.960
B0	B0	1.000	1.000
B0	A0	0.891	0.980
B0	A1	0.394	0.101
B0	A2	0.145	0.004
B0	A3	0.688	0.063
A0	B3	0.500	0.500
A0	B2	0.019	0.623
A0	B1	0.008	0.172
A0	B0	0.344	0.090
A0	A0	1.000	1.000
A0	A1	0.994	0.087

A0	A2	0.166	0.004
A0	A3	0.938	0.500
A1	B3	1.000	1.000
A1	B2	0.500	0.927
A1	B1	0.428	1.000
A1	B0	0.705	0.941
A1	A0	0.014	0.952
A1	A1	1.000	1.000
A1	A2	0.165	0.000
A1	A3	0.231	0.001
A2	B3	1.000	1.000
A2	B2	1.000	1.000
A2	B1	0.500	0.996
A2	B0	0.965	0.999
A2	A0	0.928	0.999
A2	A1	0.879	1.000
A2	A2	1.000	1.000
A2	A3	0.913	0.584
A3	B3	1.000	1.000

A3	B2	1.000	1.000
A3	B1	0.188	0.250
A3	B0	0.688	1.000
A3	A0	0.313	1.000
A3	A1	0.849	1.000
A3	A2	0.126	0.500
A3	A3	1.000	1.000

Table S8: Contingency table with number of DE genes for Fisher's exact test comparing WT with T1 (Fisher test: 0.302975206611570248; P=4.0438076214854218e-09).

	delta < 0<br>(more closed/B-like)	delta > 0<br>(more open/A-like)
lfc ≥ 2	72	132
lfc ≤ -2	132	72

Table S9: Contingency table with number of DE genes for Fisher's exact test comparing WT with C1 (Fisher test: 0.42376178720527073; P=6.6567467681675556e-13).

	delta < 0<br>(more closed/B-like)	delta > 0<br>(more open/A-like)
lfc ≥ 2	210	373
lfc ≤ -2	341	259

Table S10: P-value table computed using the Mcnemar test to assess changes of clique sizes across conditions.

**cond1**	**cond2**	**ratio**	**clique_size**	**mcnemar_stat**	**mcnemar_pval**

WT	T1	0.74	1	789	2.20E-09
WT	T1	0.58	2	690	1.30E-31
WT	T1	0.83	3	807	0.023
WT	T1	1.53	4	477	8.60E-11
WT	T1	2.27	5	241	4.70E-23
WT	T1	2	6	158	9.60E-18
WT	T1	2.38	7	60	2.70E-06
WT	T1	3.83	8	8	5.00E-15
WT	T1	inf	9	0	4.50E-13
WT	T1	inf	10	0	1
WT	T1	inf	11	0	1
WT	C1	0.91	1	770	0.65
WT	C1	0.81	2	690	0.004
WT	C1	0.76	3	807	7.30E-08
WT	C1	1.07	4	700	0.14
WT	C1	1.78	5	315	1.20E-11
WT	C1	1.6	6	225	2.10E-07
WT	C1	1.15	7	90	0.024
WT	C1	1.39	8	66	0.45

WT	C1	2	9	23	0.025
WT	C1	inf	10	0	1
WT	C1	inf	11	0	1

Table S11: Table of numbers of members in HDBSCAN clusters in 10A, T1 and C1.

cluster	MCF10A REP1	MCF10A REP2	MCF10AT1 REP1	MCF10AT1 REP2	MCF10CA1a REP1	MCF10CA1a REP2
Outliers	847	811	526	597	595	563
0	135	127	173	176	182	170
1	97	85	56	65	74	74
2	210	195	219	234	274	252
3	41	37	23	24	56	53
4	323	301	316	330	341	334
5	165	154	80	81	130	117
6	534	520	260	296	521	483
7	348	340	168	177	289	274
8	44	39	25	30	54	49

Table S12: P-value table computed using the Mcnemar test to assess changes in associations with clique clusters across conditions.

cond1	cond2	cluster	mcnemar_stat	mcnemar_pval
WT	T1	Mixed	526	6.40E-15

WT	T1	0	127	0.0093
WT	T1	1	56	0.018
WT	T1	2	195	0.26
WT	T1	3	23	0.092
WT	T1	4	301	0.57
WT	T1	5	80	1.50E-06
WT	T1	6	260	7.90E-21
WT	T1	7	168	1.90E-14
WT	T1	8	25	0.1
WT	C1	Mixed	595	9.20E-09
WT	C1	0	127	0.0021
WT	C1	1	74	0.43
WT	C1	2	195	0.00031
WT	C1	3	37	0.061
WT	C1	4	301	0.12
WT	C1	5	130	0.17
WT	C1	6	520	1
WT	C1	7	289	0.046
WT	C1	8	39	0.15

Table S13: Table of differentially expressed genes in T1 compared to 10A (lfc>= 0.1, svalue<0.01)

Gene name	chrom	start	end	log2FoldChange	svalue
LRATD2	chr8	126552443	126558478	1.53	4.32E-160
CASC19	chr8	127072694	127227541	-0.36	1.16E-06
CASC8	chr8	127289808	127482139	1.16	7.19E-24
ENSG00000286010	chr8	127663280	127670990	-1.32	0.00E+00
PVT1	chr8	127794526	128187101	0.28	2.95E-04
UNC5B	chr10	71212570	71302864	0.62	2.66E-09
SLC29A3	chr10	71319259	71381423	0.65	1.02E-05
VSIR	chr10	71747556	71773520	0.90	1.33E-26
PSAP	chr10	71816298	71851251	0.59	7.25E-12
CHST3	chr10	71964395	72013558	0.69	9.67E-15
SPOCK2	chr10	72059034	72089032	1.53	7.87E-06
ASCC1	chr10	72096032	72217134	1.15	5.94E-71
ANAPC16	chr10	72216000	72235860	1.09	1.19E-36
ENSG00000289506	chr10	72272288	72273704	2.36	6.49E-04
DDIT4	chr10	72273919	72276036	1.45	7.05E-127
DNAJB12	chr10	72332830	72355149	0.71	3.01E-36
MICU1	chr10	72367340	72626131	1.03	3.08E-59

MCU	chr10	72692143	72887694	1.36	2.49E-133
P4HA1	chr10	73007217	73096974	0.36	1.27E-05
NUDT13	chr10	73110375	73131828	1.96	7.12E-30
ENSG00000272599	chr10	73124573	73125532	1.45	4.49E-12
ECD	chr10	73130155	73169055	1.30	3.06E-105
FAM149B1	chr10	73168119	73244504	0.66	3.85E-21
ENSG00000288559	chr10	73247342	73248268	0.87	1.71E-05
MRPS16	chr10	73248843	73252693	0.42	2.03E-17
DNAJC9-AS1	chr10	73252791	73254349	1.40	3.09E-11

Table S14: Table of differentially expressed genes in C1 compared to 10A (lfc>= 0.1, svalue<0.01)

Gene name	chrom	start	end	log2FoldChange	svalue
LRATD2	chr8	126552443	126558478	2.09	1.62E-304
PCAT1	chr8	126556323	127419050	2.11	1.13E-17
CASC19	chr8	127072694	127227541	0.86	1.38E-36
ENSG00000224722	chr8	127086263	127087510	2.13	1.26E-05
ENSG00000287781	chr8	127253213	127257630	3.13	7.17E-08
CASC8	chr8	127289808	127482139	2.90	1.54E-150
POU5F1B	chr8	127322183	127420066	2.95	4.37E-53

ENSG00000286010	chr8	127663280	127670990	-0.41	1.98E-04
CASC11	chr8	127686343	127738987	1.92	1.45E-05
MYC	chr8	127735434	127742951	0.25	1.86E-04
PVT1	chr8	127794526	128187101	1.67	1.57E-122
UNC5B	chr10	71212570	71302864	3.43	1.44E-266
UNC5B-AS1	chr10	71217220	71218294	1.33	2.33E-04
SLC29A3	chr10	71319259	71381423	0.74	6.98E-07
CDH23	chr10	71396920	71815947	1.77	1.40E-04
VSIR	chr10	71747556	71773520	3.25	0.00E+00
PSAP	chr10	71816298	71851251	3.09	0.00E+00
ENSG00000289592	chr10	71888499	71889171	3.21	1.39E-10
CHST3	chr10	71964395	72013558	1.03	2.71E-32
SPOCK2	chr10	72059034	72089032	4.04	5.13E-29
ASCC1	chr10	72096032	72217134	2.03	8.81E-228
ANAPC16	chr10	72216000	72235860	1.22	4.50E-46
ENSG00000289506	chr10	72272288	72273704	6.13	8.21E-14
DDIT4	chr10	72273919	72276036	2.14	6.95E-284
DNAJB12	chr10	72332830	72355149	0.77	5.06E-43
MICU1	chr10	72367340	72626131	1.09	2.50E-64

MCU	chr10	72692143	72887694	2.08	0.00E+00
P4HA1	chr10	73007217	73096974	2.29	6.14E-203
NUDT13	chr10	73110375	73131828	2.63	1.71E-51
ECD	chr10	73130155	73169055	2.22	0.00E+00
FAM149B1	chr10	73168119	73244504	1.78	8.33E-158
DNAJC9	chr10	73183362	73247255	0.46	2.33E-12
ENSG00000288559	chr10	73247342	73248268	1.94	6.42E-19
MRPS16	chr10	73248843	73252693	0.50	2.83E-25
DNAJC9-AS1	chr10	73252791	73254349	1.87	5.05E-18

Table S15: List of manually annotated translocations for 10A.

**chrom1**	**start1**	**end1**	**chrom2**	**start2**	**end2**
chr3	894000	68025000	chr9	23591000	138394717
chr3	1235000	68434000	chr5	118992000	181538259
chr3	68025000	198295559	chr9	0	20878000
chr5	118960000	181538259	chr9	23908000	138394717

Table S16: List of manually annotated translocations for T1.

**chrom1**	**start1**	**end1**	**chrom2**	**start2**	**end2**
chr3	0	59225000	chr17	16340000	83257441
chr3	894000	68025000	chr9	23591000	138394717
chr3	1235000	68434000	chr5	118992000	181538259
chr3	56933000	90650000	chr17	0	19300000
chr3	68025000	198295559	chr9	0	20878000
chr5	118960000	181538259	chr9	23908000	138394717
chr6	0	170805979	chr19	33715000	58617616
chr8	126000000	128300000	chr10	0	133797422

Table S17: List of manually annotated translocations for C1.

**chrom1**	**start1**	**end1**	**chrom2**	**start2**	**end2**
chr2	0	92100000	chr10	41600000	133797422

chr3	0	59225000	chr17	16340000	83257441
chr3	0	68462000	chr7	0	150000000
chr3	894000	68025000	chr9	23591000	138394717
chr3	1235000	68434000	chr5	118992000	181538259
chr3	56933000	90650000	chr17	0	19300000
chr3	68025000	198295559	chr9	0	20878000
chr5	118960000	181538259	chr9	23908000	138394717
chr6	0	170805979	chr19	33715000	58617616
chr7	63700000	151700000	chr9	24123000	35780000
chr8	126000000	128300000	chr10	0	133797422
chr10	0	74500000	chr17	29970000	83257441

Table S18: Number of nuclei and probes detected from FISH microscopy data. (A=*MYC* probe; D=enhancer probe; E=Distal non-enhancer probe.)

	**10A (AD)**	**10A (AE)**	**C1 (AD)**	**C1 (AE)**
**num_valid_nuclei**	34	28	95	43
**num_nuclei**	45	37	129	70
**num_red_probes**	62	62	269	140
**num_green_probes**	61	60	272	86
