# Supplementary material for: Multi-level 3D genome organization deteriorates during breast cancer progression
Source: bioRxiv. 2023 Nov 27:2023.11.26.568711. Preprint. [Version 1] doi: 10.1101/2023.11.26.568711 (PMC10705249; doi:10.1101/2023.11.26.568711)
Supplement: Supplementary file 1 [file media-1.pdf]

Multi-level 3D genome organization deteriorates during breast cancer progression

Supplementary figures

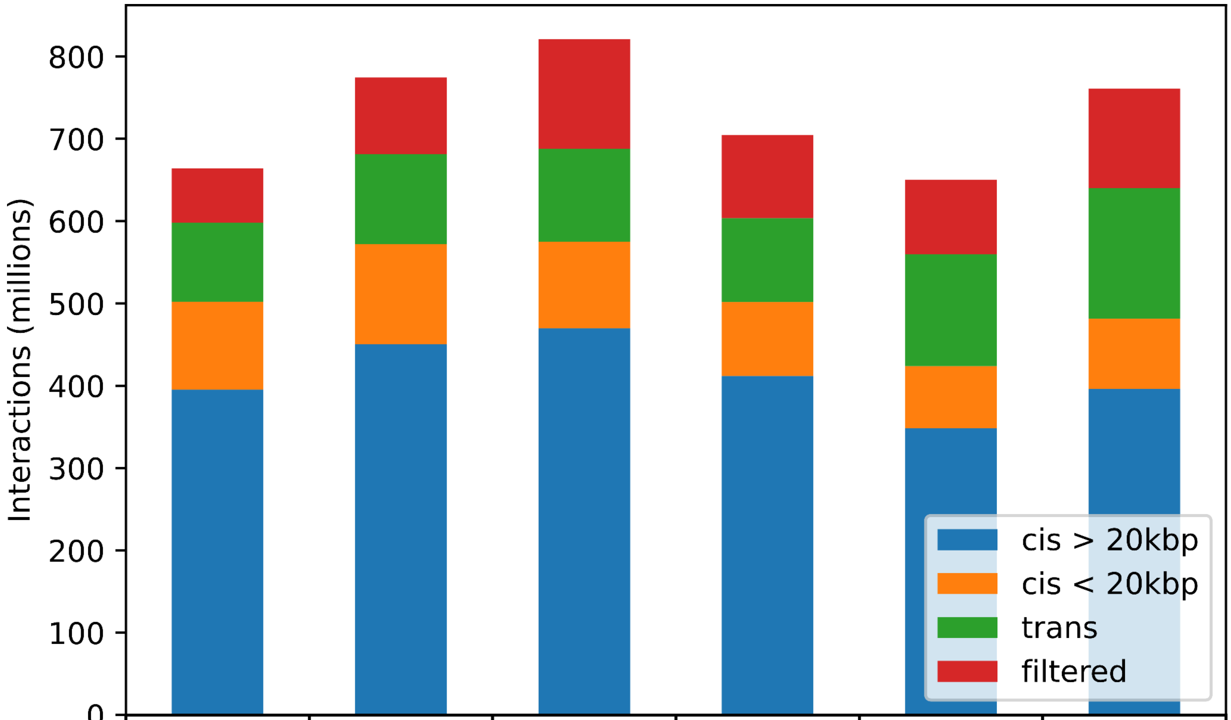

	MCF10A rep.1	MCF10A rep.2	MCF10AT1 rep.1	MCF10AT1 rep.2	MCF10CA1a rep.1	MCF10CA1a rep.2
cis > 20kbp	395.19	450.37	469.72	411.8	348.31	396.13
cis < 20kbp	106.91	121.64	105.22	89.85	75.64	85.27
trans	95.93	109.5	112.73	101.96	135.7	158.56
filtered	66.14	92.97	133.61	101.12	90.5	120.95
total	664.17	774.48	821.28	704.73	650.15	760.91

Fig. S1: Barplot showing the number of Hi-C interactions (in millions) classified by type: long-range cis (blue), short-range cis (orange), trans (green) and filtered out (red). The table under the graph shows the data underlying the barplot.

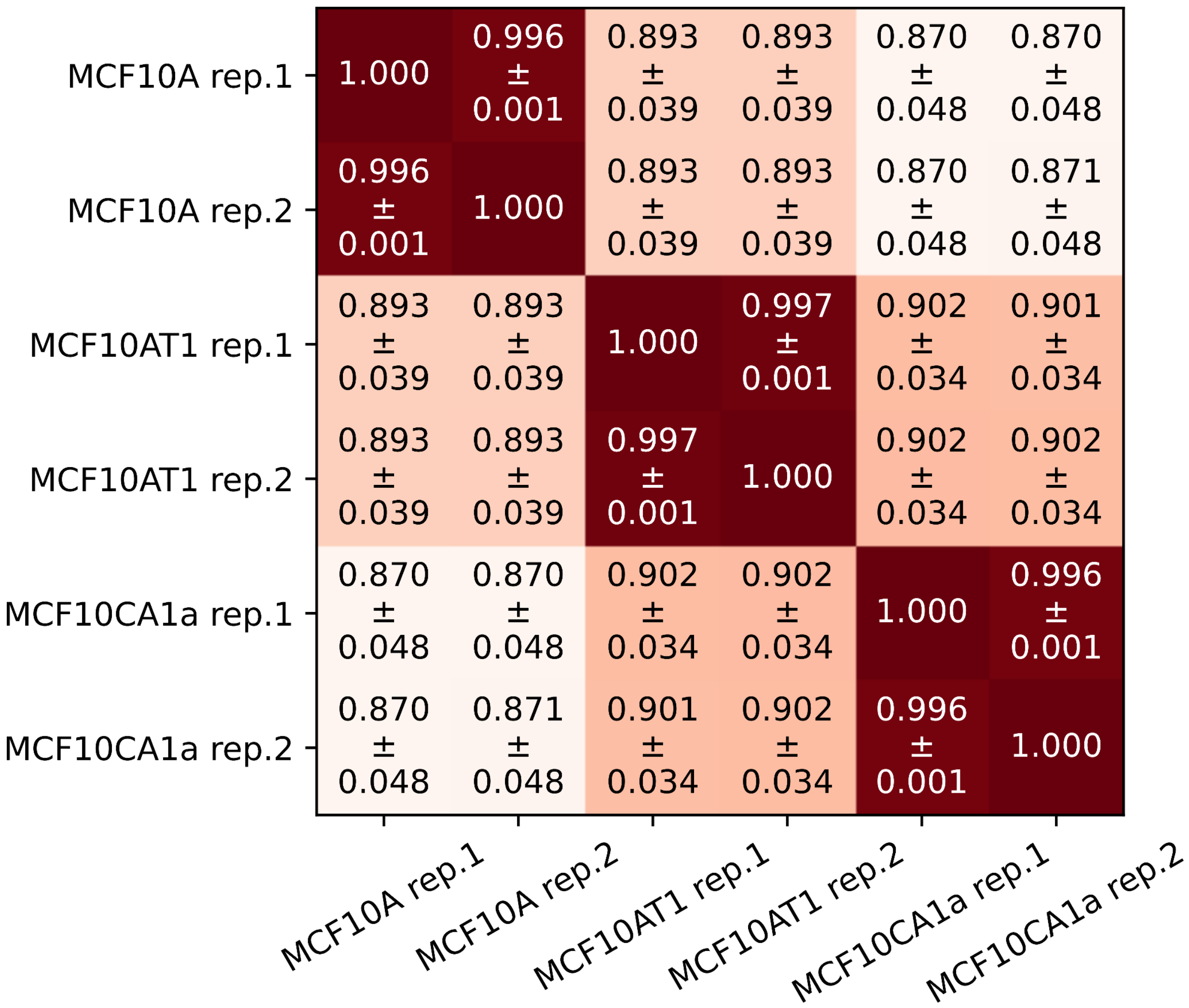

Fig. S2: Heatmap showing the weighted average of the stratified correlation coefficient (SCC) computed for each pair of samples. Chromosome sizes are used as weights in the computation of the SCC weighted average. Overlaid numbers show the weighted average and standard deviation for each pair of samples.

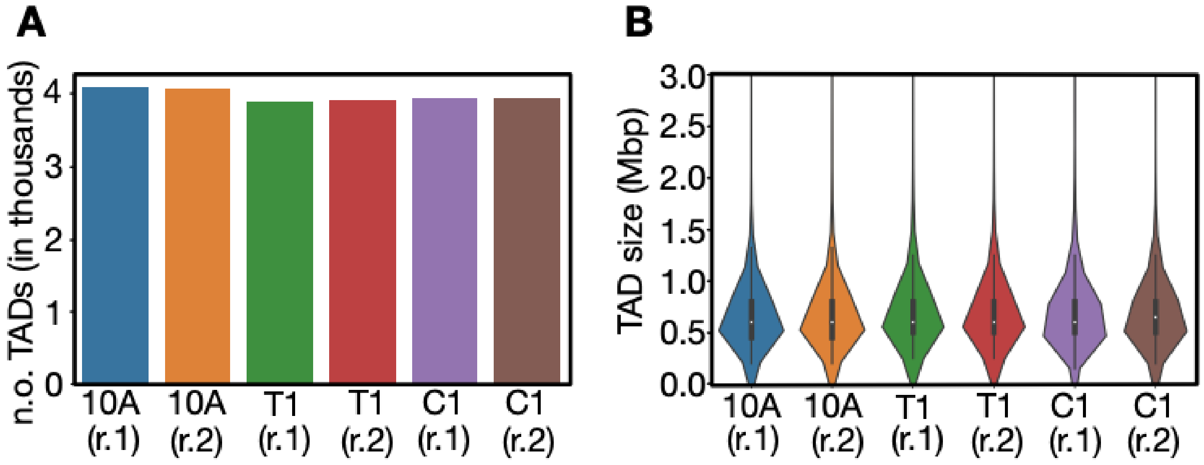

Fig. S3: **A**: Number of TADs (in thousands) for each of the six Hi-C samples generated. **B**: Comparison of genomic sizes of all TADs for the six samples.

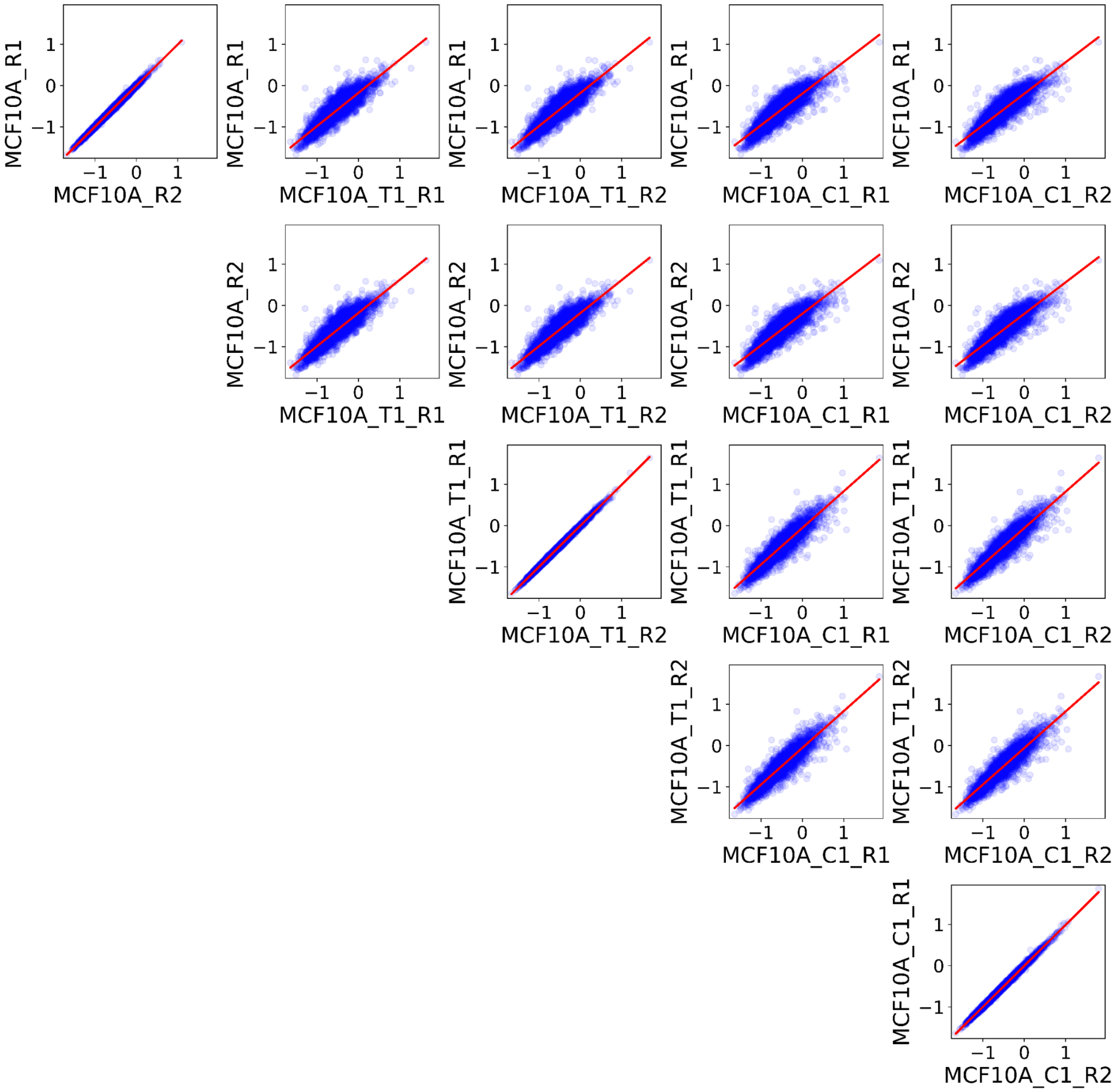

Fig. S4: Scatterplot contrasting TAD insulation scores at 50 kbp resolution for all possible pairs of samples. Trend line is depicted in red.

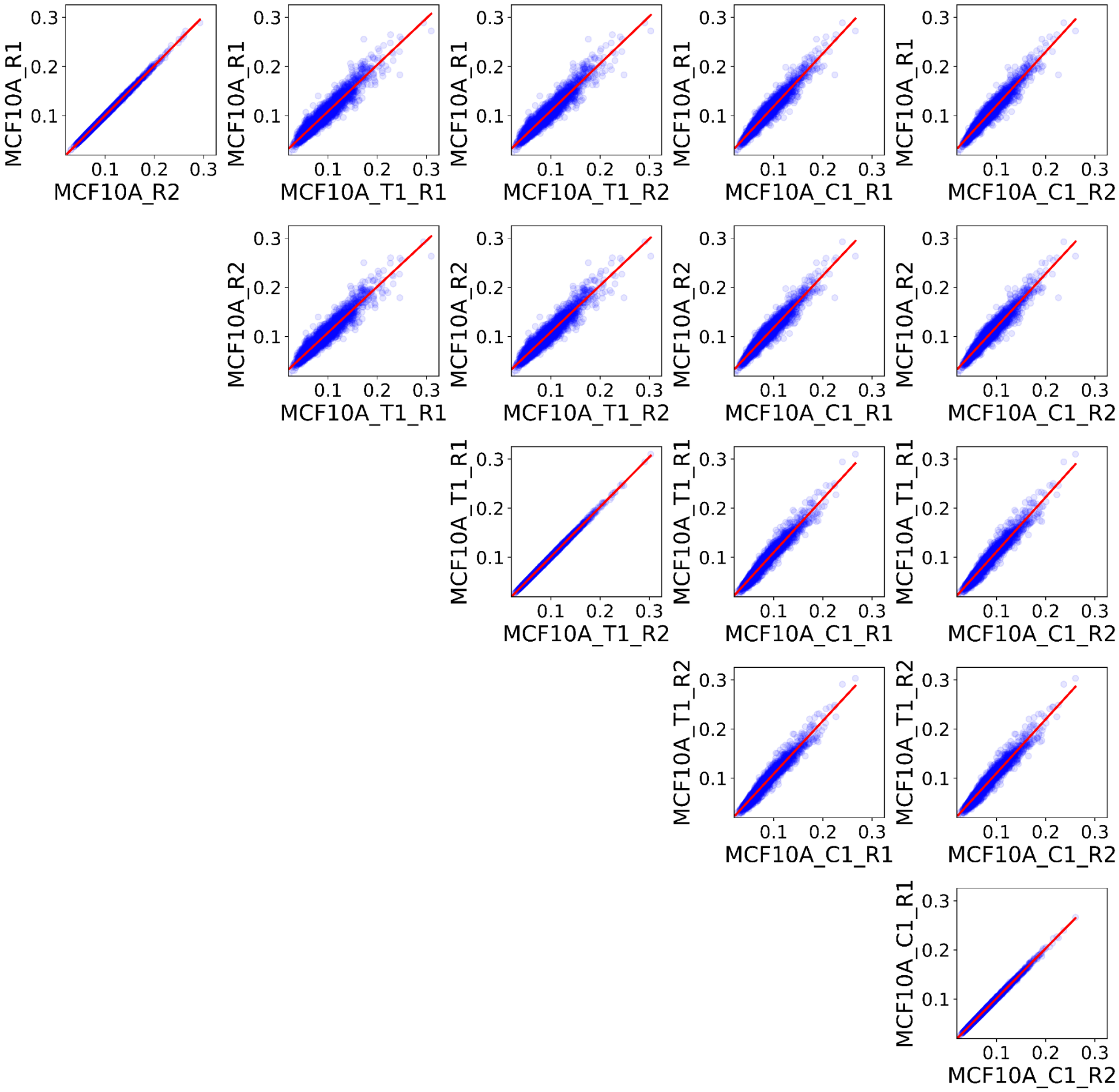

Fig. S5: Scatterplot contrasting the normalized number of interactions of MCF10A TADs at 50 kbp resolution across all possible pairs of samples. Trend line is shown in red. Scores are computed by aggregating interactions from the upper triangle of the Hi-C matrix overlapping TADs. Aggregated interactions are normalized based on the number of pixels belonging to a TAD.

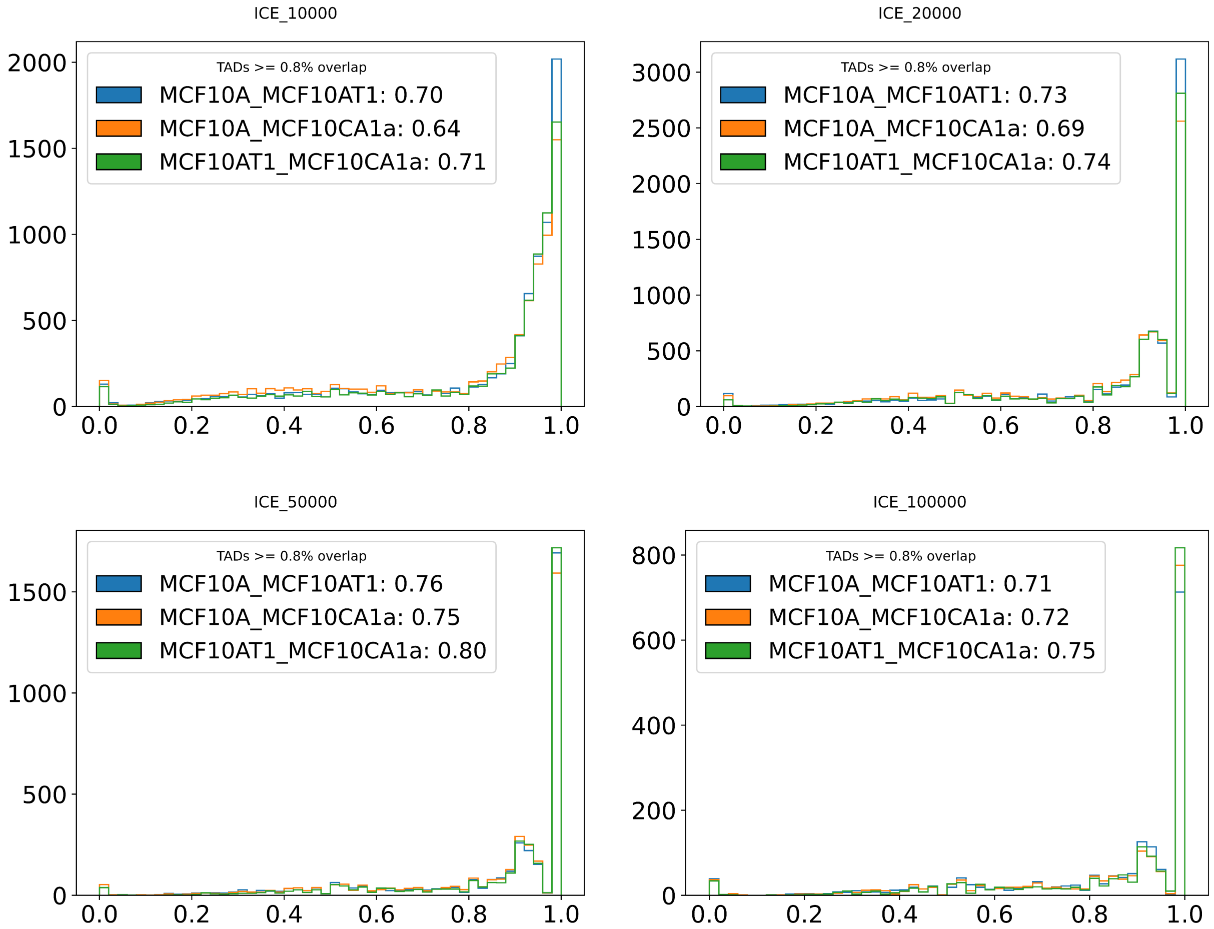

Fig. S6: Histogram of TAD Jaccard overlap coefficients. Coefficients are computed from Hi-C matrices after collapsing replicates. Numbers shown in plot legends show the fraction of TADs with at least 80% overlap. Panels show coefficients computed using TADs called at 10 kbp (top left), 20 kbp (top right), 50 kbp (bottom left) and 100 kbp (bottom right) resolutions.

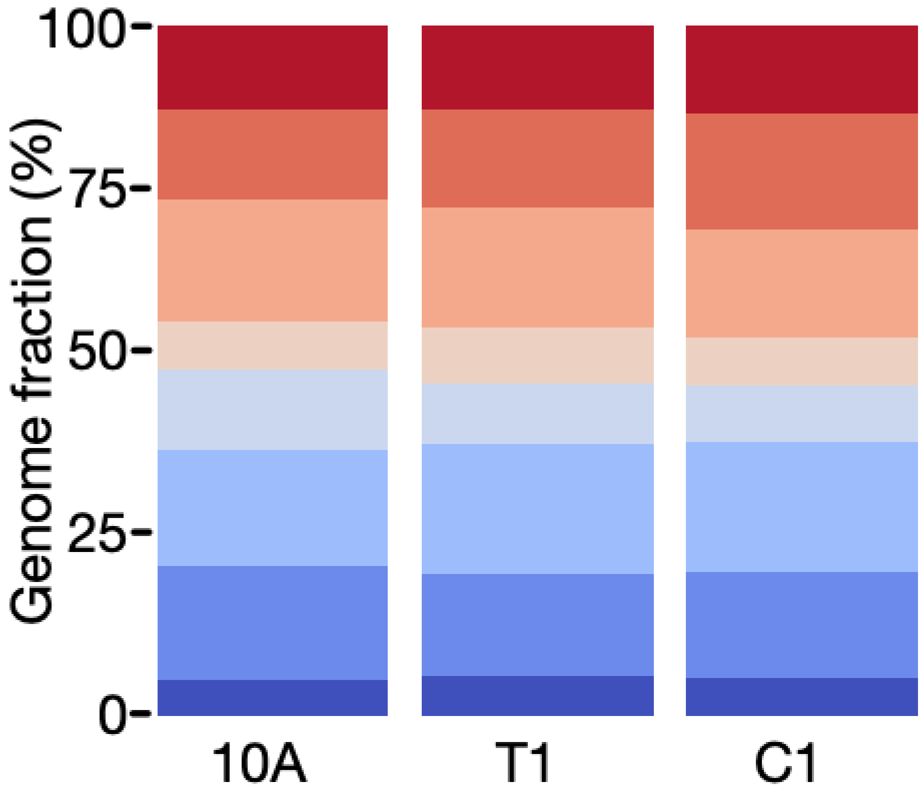

Fig. S7: Relative genome coverage of subcompartments (at 10kbp resolution) in the three samples.

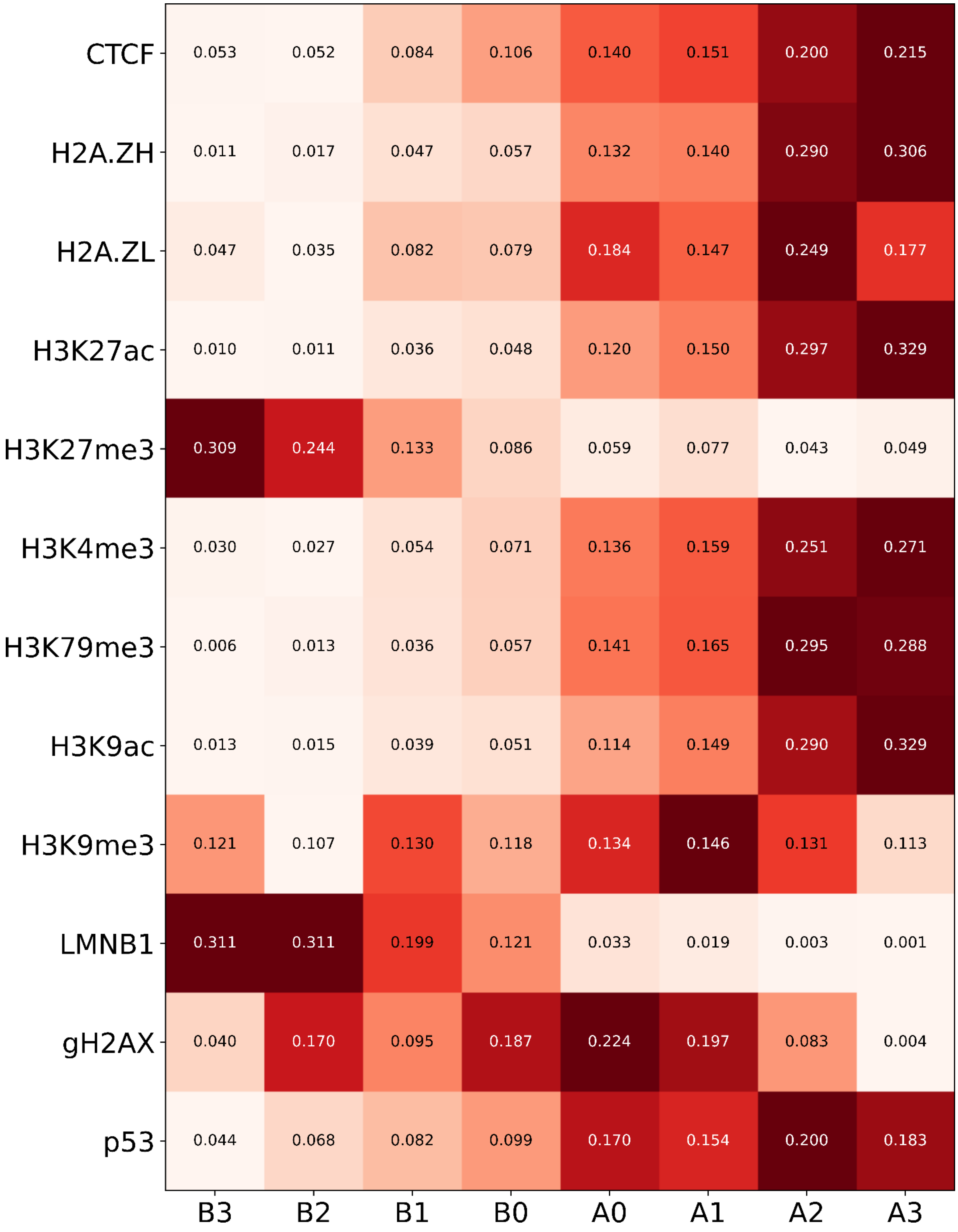

Fig. S8. Heatmap showing the average coverage of several epigenetic marks across subcompartments called on MCF10A matrices after collapsing replicates at 10 kbp resolution. Values overlaid on the heatmap show the average intensity of ChIP-seq signal over peaks overlapping each subcompartment. Color scale is normalized separately for each row to show the lowest and highest values of each row in white and dark red respectively.

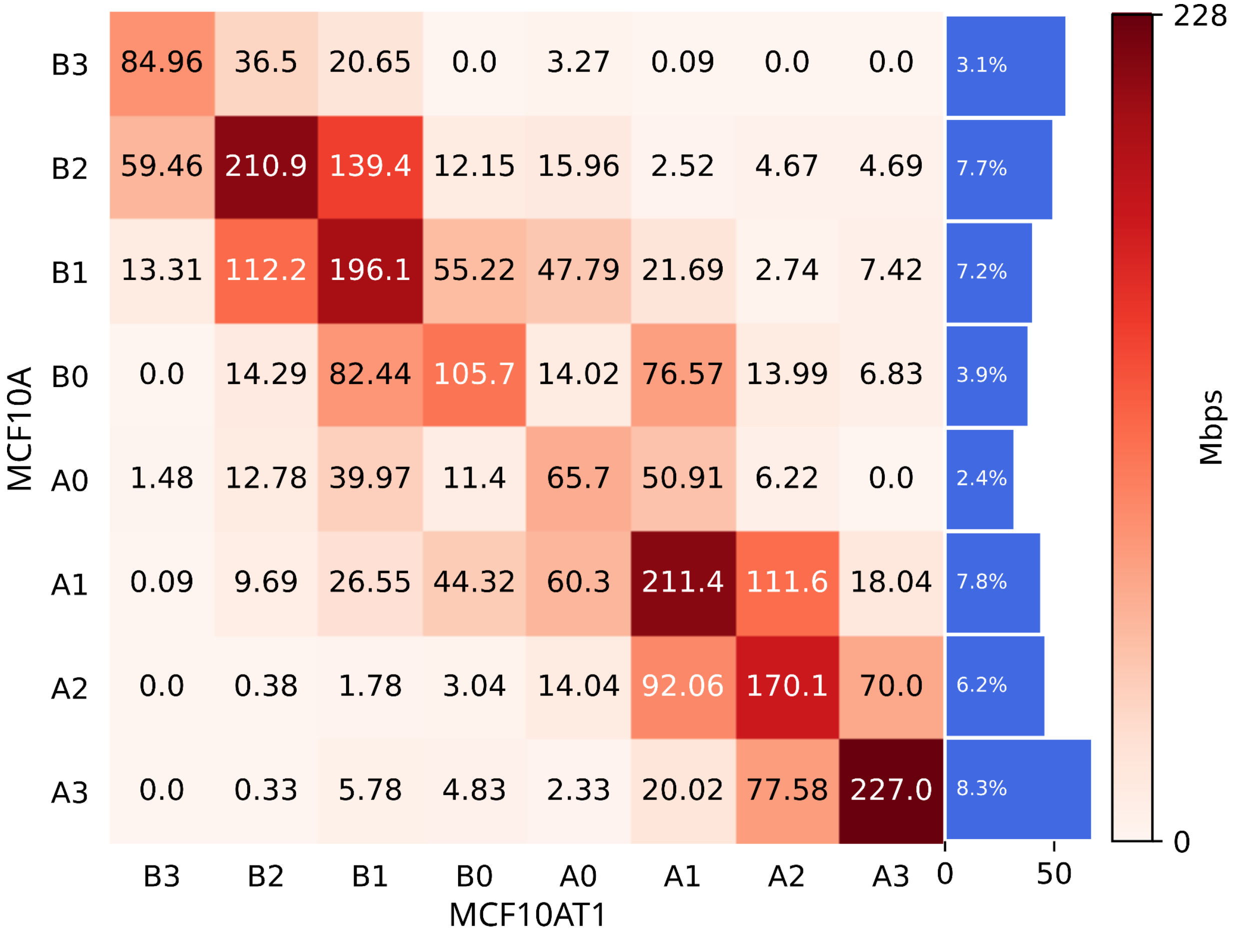

Fig. S9: Subcompartment switches (10kbp resolution) for MCF10A (WT) (vertical axis) vs MCF10AT1 (T1) (horizontal axis). Numbers are expressed in Mbp unless otherwise specified. Subcompartment switches are computed by comparing subcompartment labels across cell types. Comparison is done at the bin-level, comparing the same genomic regions.
The bar plot shows the fraction of Mbps not involved in subcompartment switching. Bars are relative to the number of Mbps belonging to a given subcompartment in at least one cell type. The percentage shows the fraction of Mbps not involved in subcompartment switching relative to the entire matrix.

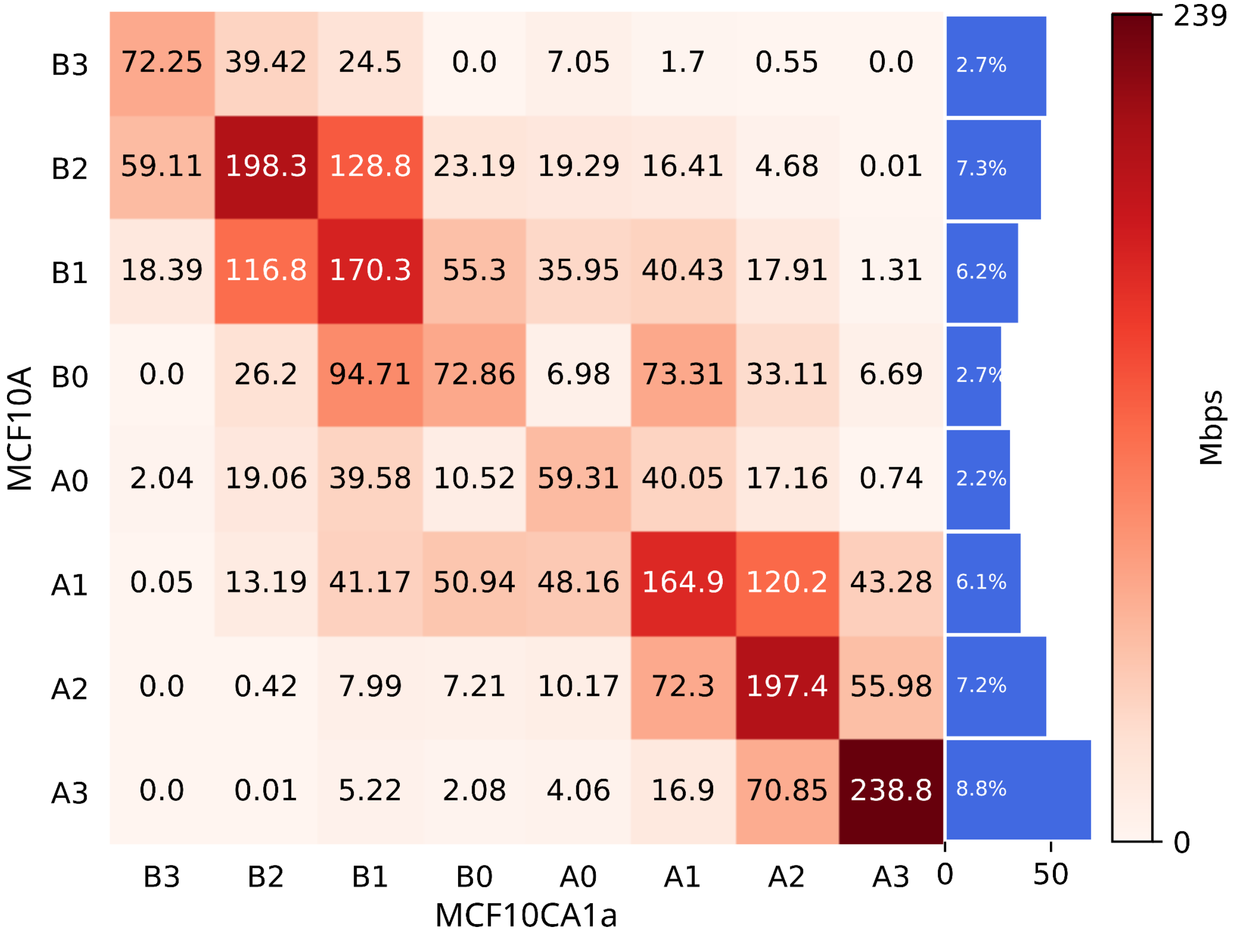

Fig. S10: Subcompartment switches (10kbp resolution) for MCF10A (WT) (vertical axis) vs MCF10CA1a (C1) (horizontal axis). Numbers are expressed in Mbp unless otherwise specified. Subcompartment switches are computed by comparing subcompartment labels across cell types. Comparison is done at the bin-level, comparing the same genomic regions. The bar plot shows the fraction of Mbps not involved in subcompartment switching. Bars are relative to the number of Mbps belonging to a given subcompartment in at least one cell type. The percentage shows the fraction of Mbps not involved in subcompartment switching relative to the entire matrix.

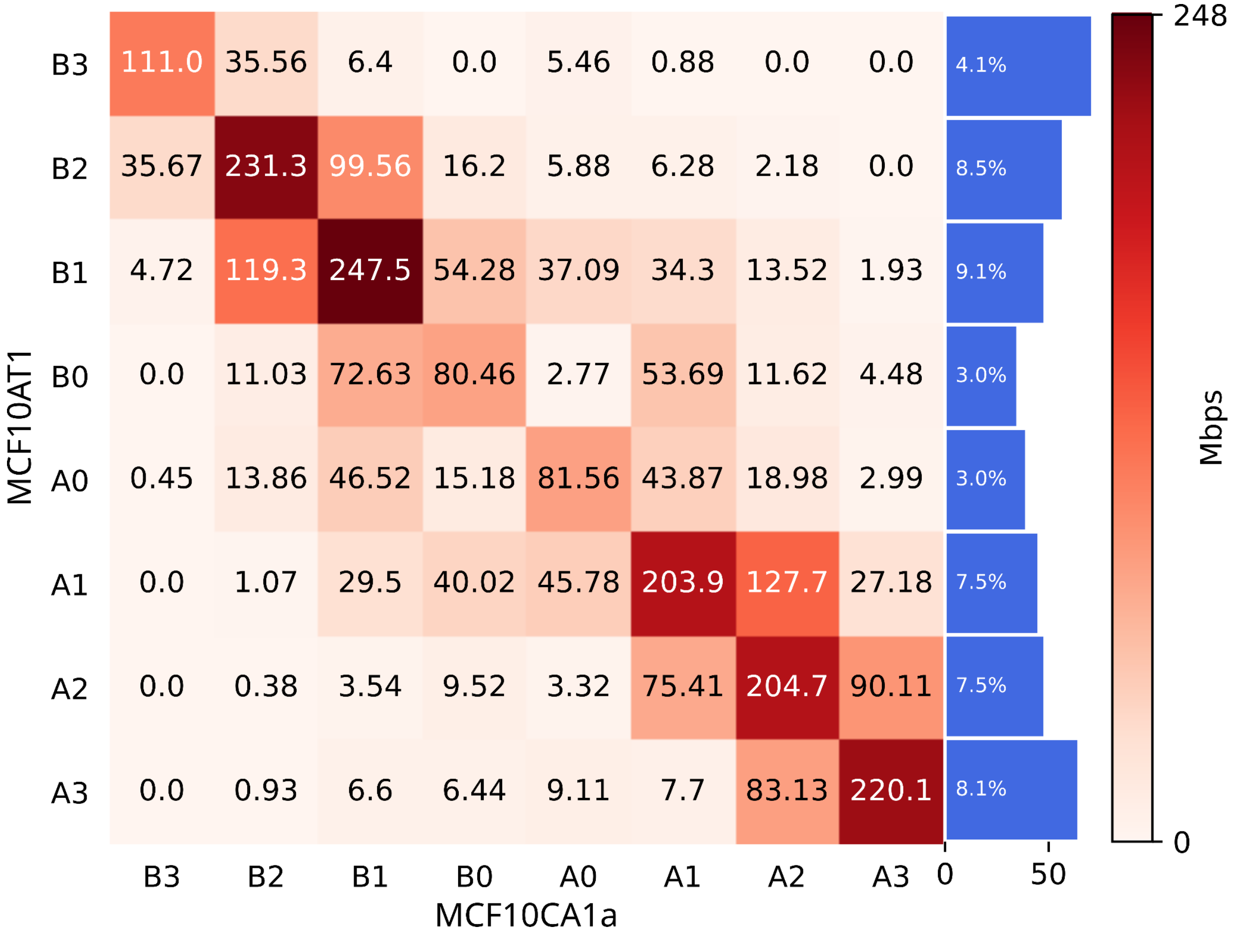

Fig. S11: Subcompartment switches (10kbp resolution) for MCF10AT1 (T1) (vertical axis) vs MCF10CA1a (C1) (horizontal axis). Numbers are expressed in Mbp unless otherwise specified. Subcompartment switches are computed by comparing subcompartment labels across cell types. Comparison is done at the bin-level, comparing the same genomic regions. The bar plot shows the fraction of Mbps not involved in subcompartment switching. Bars are relative to the number of Mbps belonging to a given subcompartment in at least one cell type. The percentage shows the fraction of Mbps not involved in subcompartment switching relative to the entire matrix.

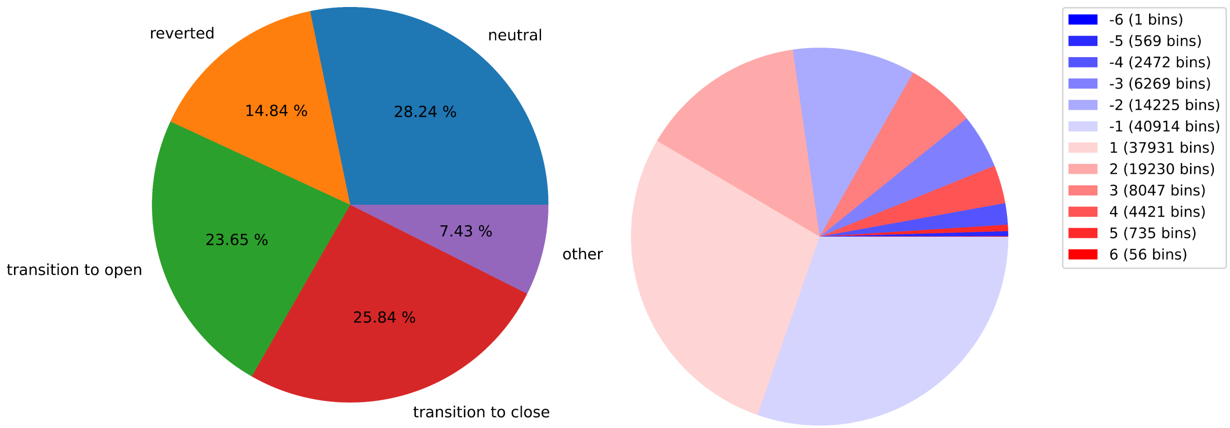

Fig. S12:
Left: Pie chart showing the fate of genomic regions involved in subcompartment switches from WT via T1 to C1. Right: Pie chart showing the magnitude and direction of subcompartment switching for genomic regions involved in subcompartment switches. Positive numbers indicate a switch towards A-like subcompartments while negative values represent a switch towards B-like subcompartments. Values represent the number of consecutive subcompartment steps that are switched (e.g. A3→A0 gives a value of -3).

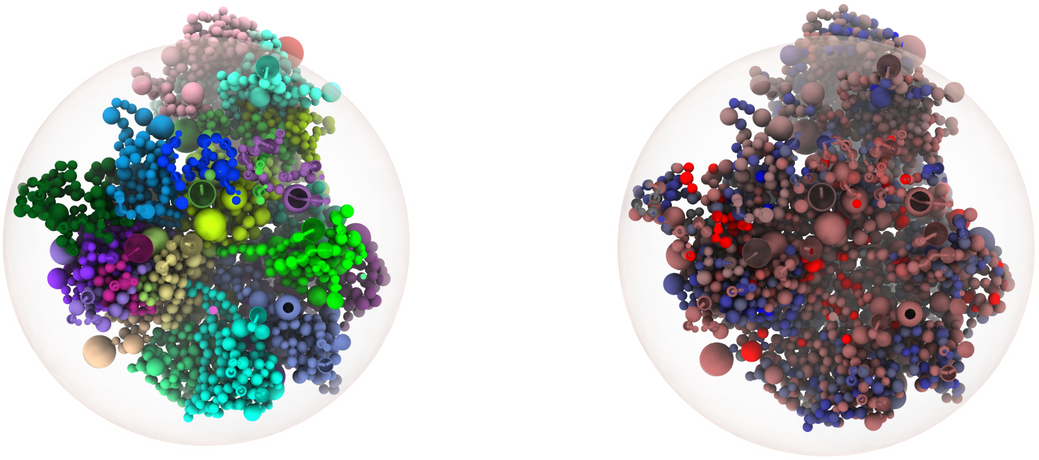

Fig S13: Left: Exemplary Chrom3D simulation model for 10A cells. Beads represent TADs sized according to genomic coverage. Chromosomes are colored distinctly. Right: The same model using sub-compartments for coloring each bead. The relative size of the model nucleus is indicated with a transparent sphere.

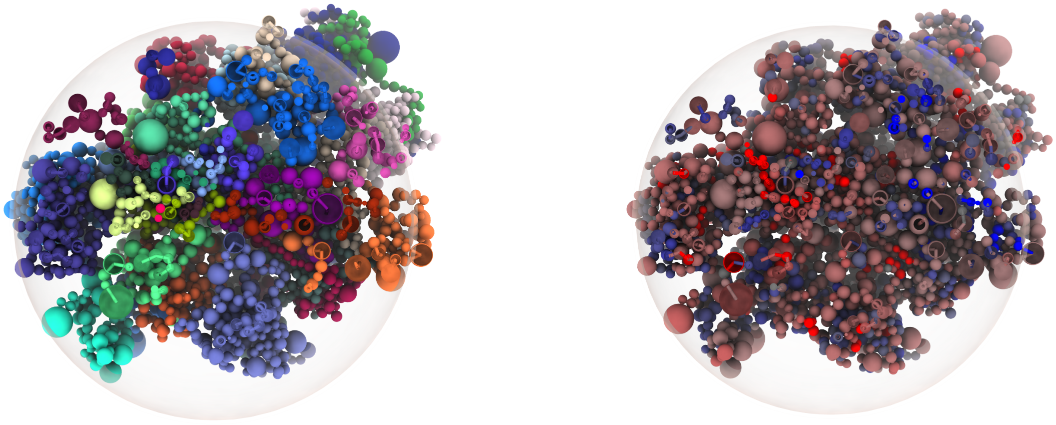

Fig S14: Left: Exemplary Chrom3D simulation model for T1 cells. Beads represent TADs sized according to genomic coverage. Chromosomes are colored distinctly. Right: The same model using sub-compartments for coloring each bead. The relative size of the model nucleus is indicated with a transparent sphere.

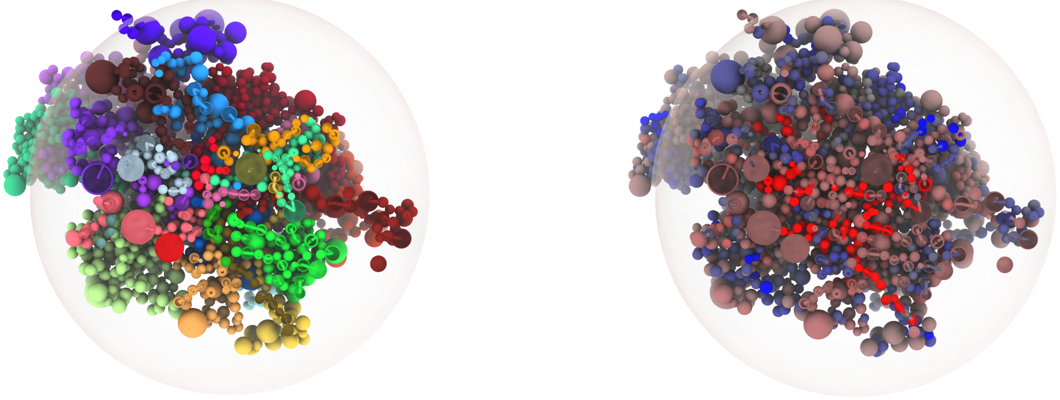

Fig S15: Left: Exemplary Chrom3D simulation model for C1 cells. Beads represent TADs sized according to genomic coverage. Chromosomes are colored distinctly. Right: The same model using sub-compartments for coloring each bead. The relative size of the model nucleus is indicated with a transparent sphere.

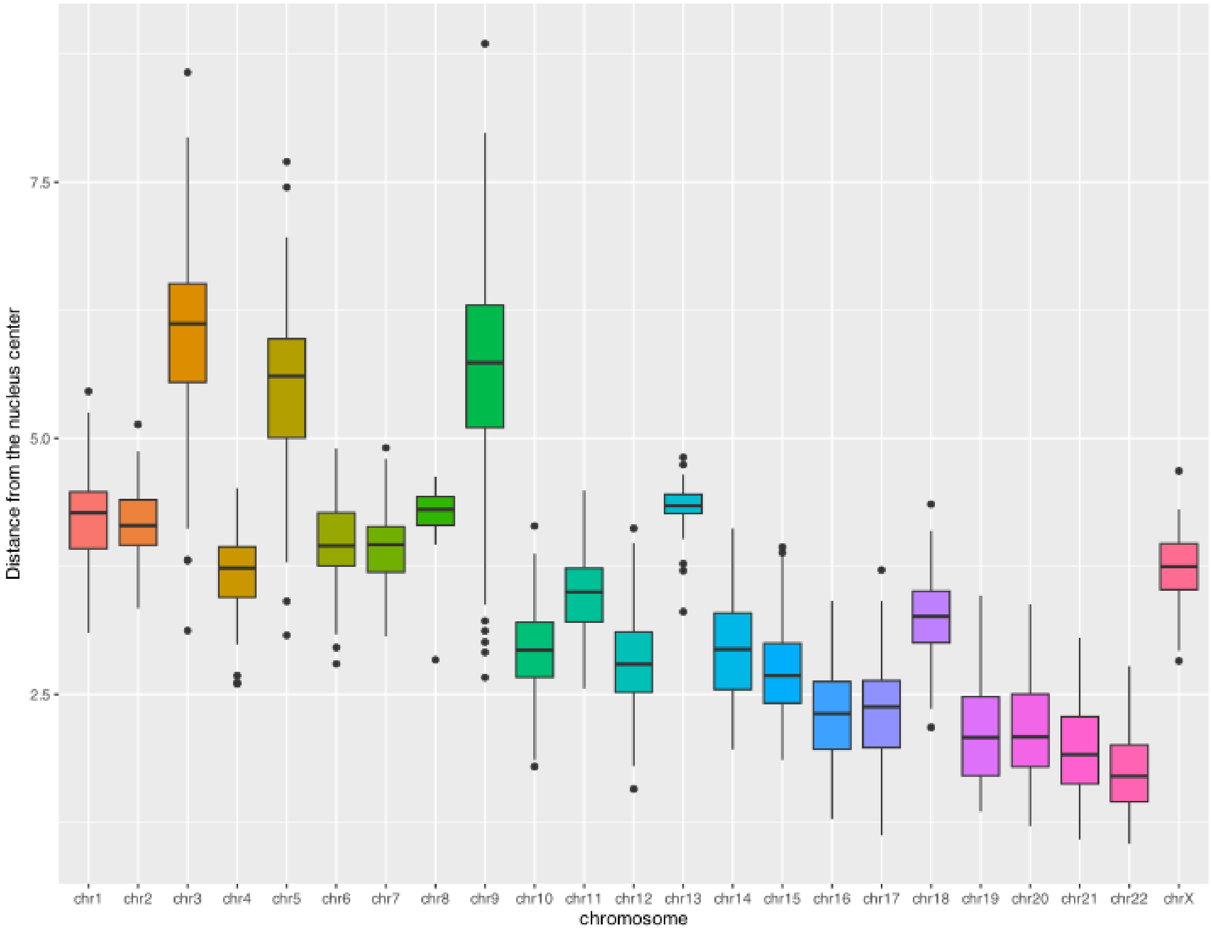

Fig. S16: Median chromosome distance from the nucleus center in 100 Chrom3D simulation models based on 10A.

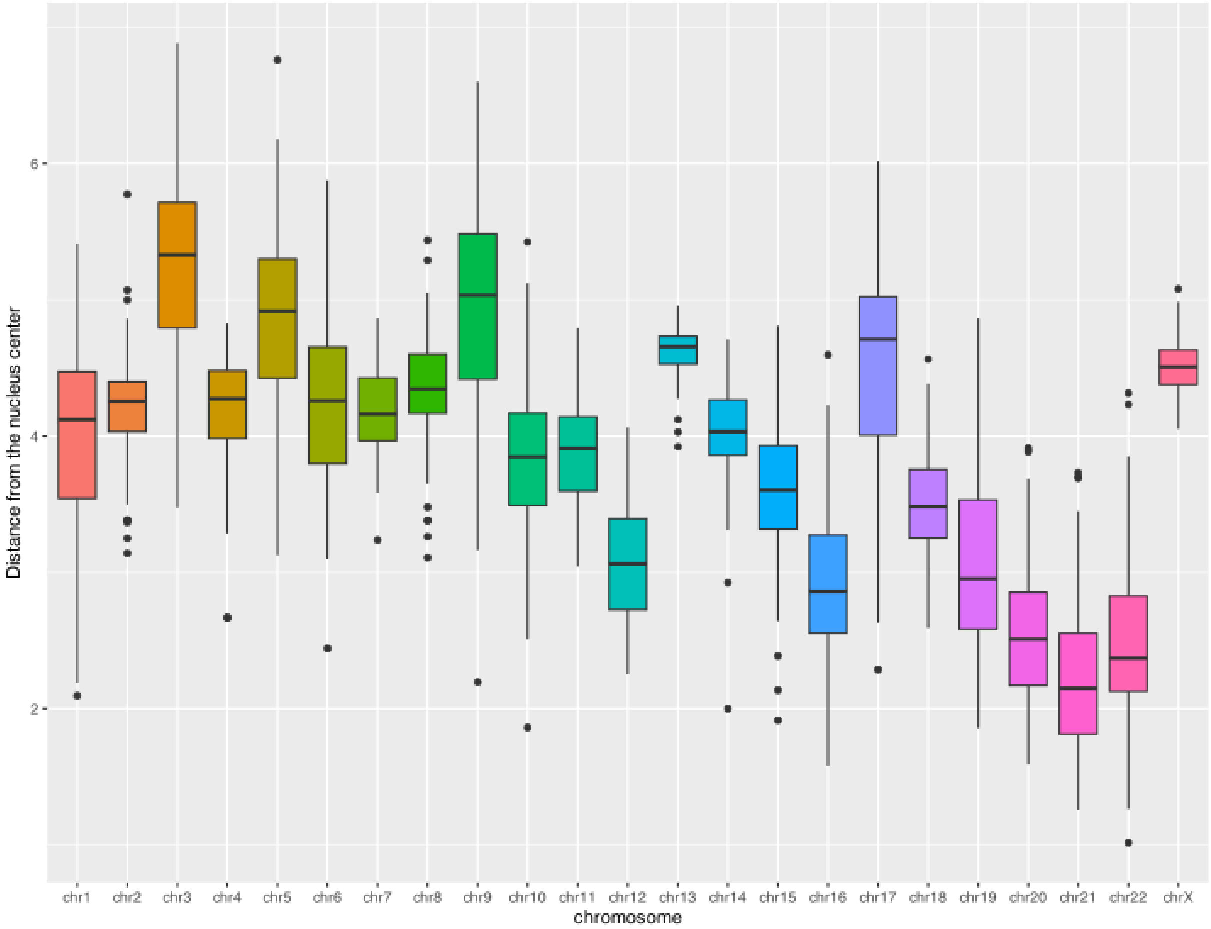

Fig. S17: Median chromosome distance from the nucleus center in 100 Chrom3D simulation models based on T1.

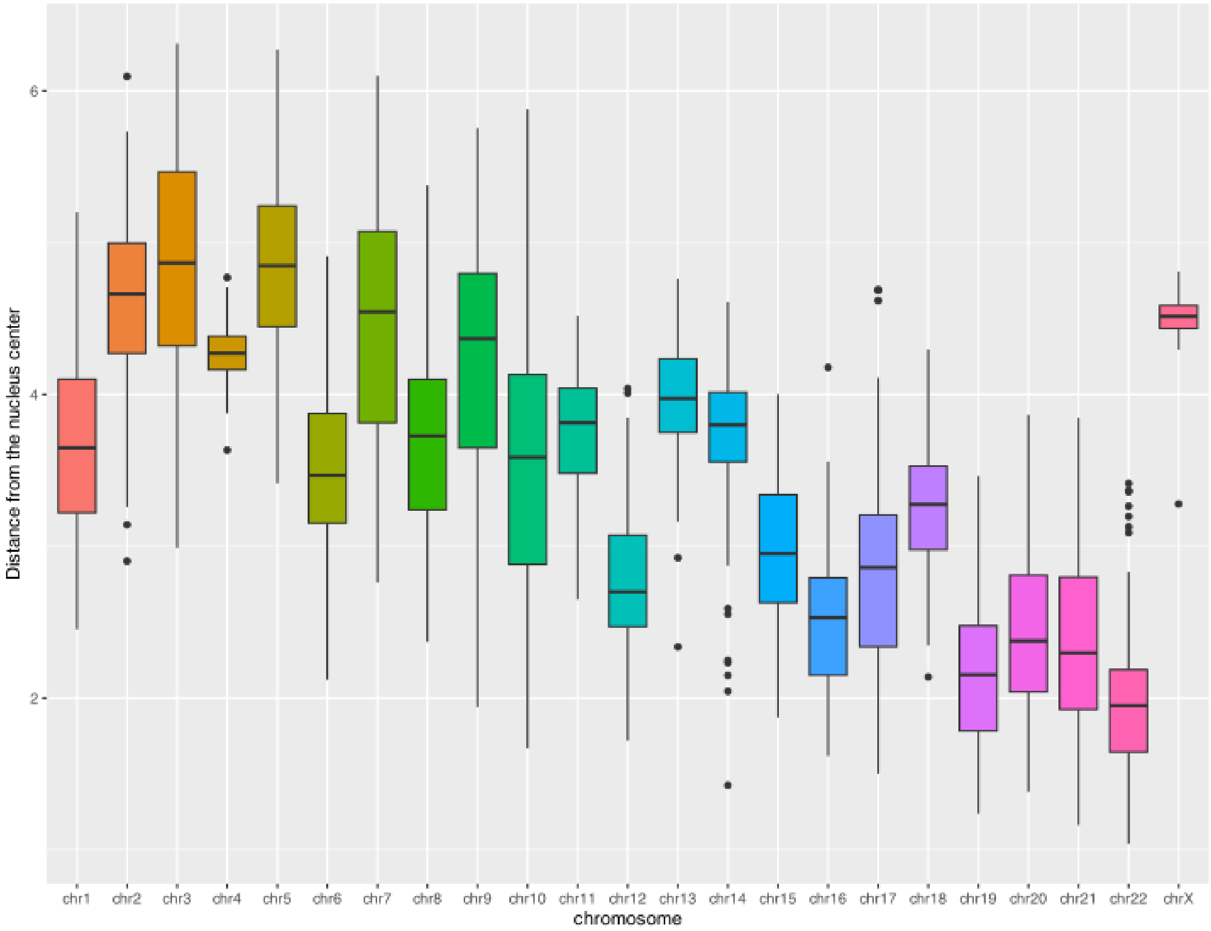

Fig. S18: Median chromosome distance from the nucleus center in 100 Chrom3D simulation models based on C1.

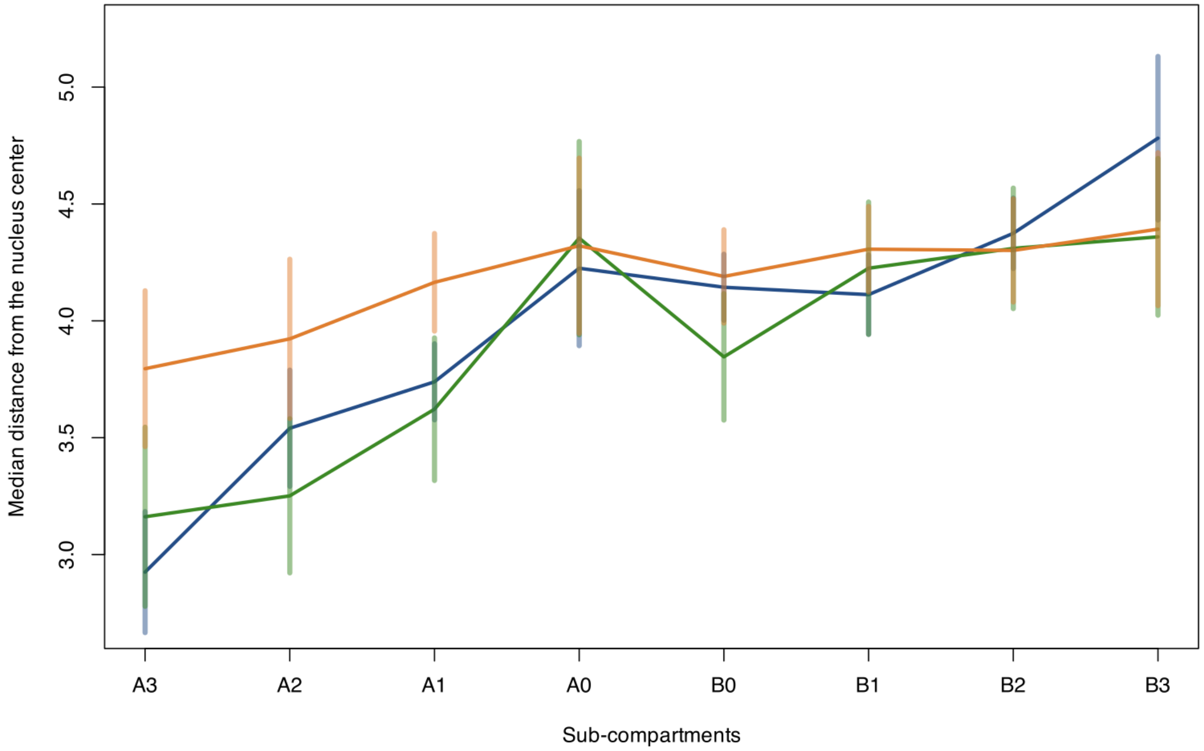

Fig. S19: Plot of the median distance from the nuclear center and the standard deviation (vertical lines) for each subcompartment in each condition in a total of 100 Chrom3D simulation models per condition. 10A is shown in blue, T1 is shown in yellow, C1 is shown in green.

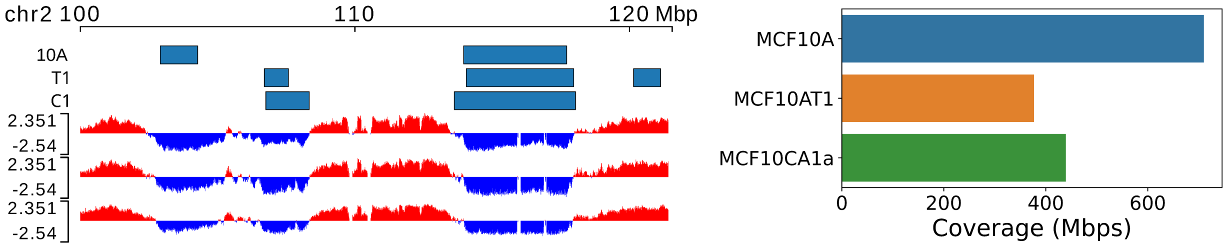

Fig. S20: Coverage of lamina associated domains (LADs).
Left: browser view of a region on chromosome 2 showing LMNB1 LADs for MCF10A (WT), MCF10AT1 (T1) and MCF10CA1a (C1) as blue bars. Principal component 1 for the same cell lines is shown below. Regions corresponding to A and B compartments are colored in red and blue respectively. Right: Genome-wide coverage of lamina associated domains.

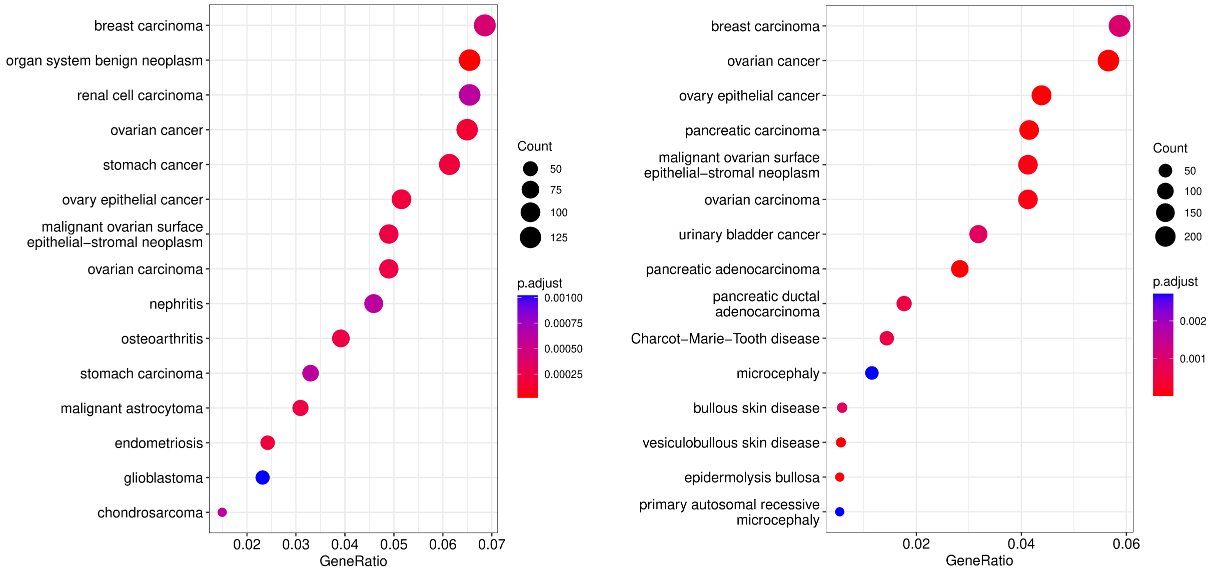

Fig. S21: Over-representation analysis for disease ontology (DO) terms for differentially expressed genes in MCF10AT1 (T1) (left) and MCF10CA1a (C1) (right) using MCF10A (10A) as contrast (lfc=0.5, p-value=0.01). Disease terms are sorted by the fraction of differentially expressed genes found in a given gene set. Dot size is proportional to the number of differentially expressed genes found in a given gene set. Dots are colored based on the statistical significance of the enrichment.

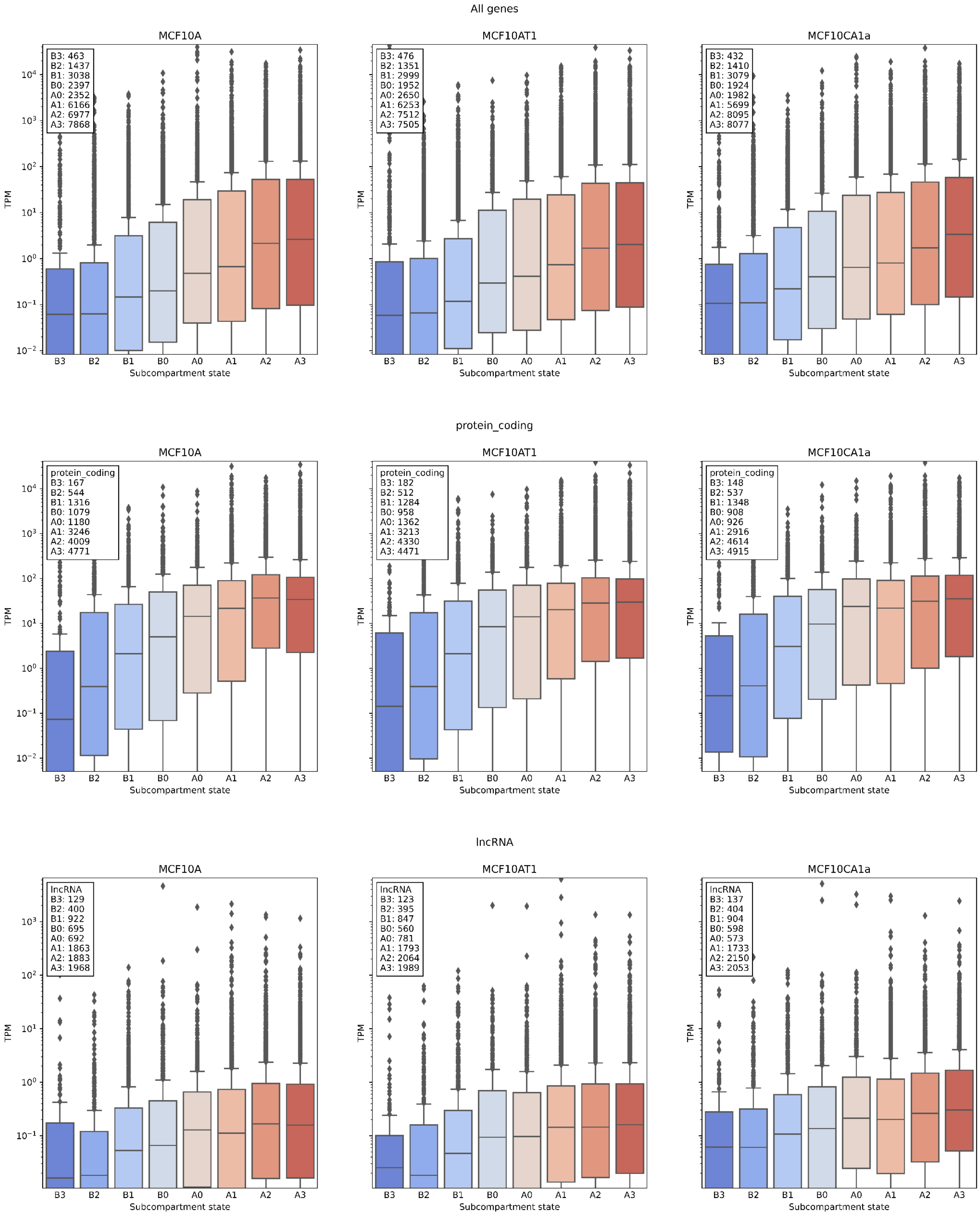

Fig. S22 Distribution of expression levels (TPM) for each subcompartment across MCF10A (left panels), MCF10AT1 (middle panels) and MCF10CA1a (right panels). Rows 1 to 3 show the expression level for all genes, genes encoding for proteins, and long non-coding RNA, respectively. Legend shows the number of genes expressed in each subcompartment.

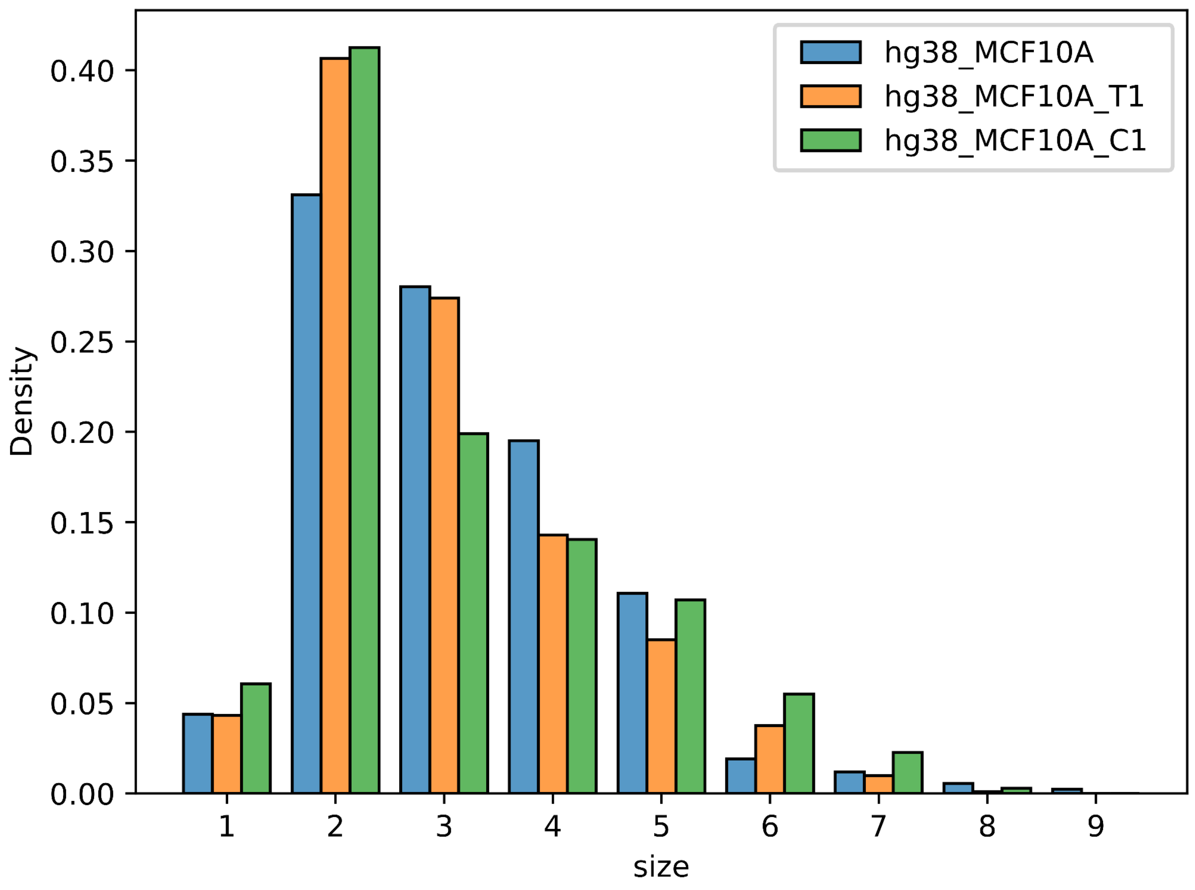

Fig. S23: Barplot showing the distribution of maximal clique sizes for MCF10A (blue), MCF10AT1 (orange) and MCF10CA1a (green). Heights of bars are normalized such that bars for each cell type sum up to 1.

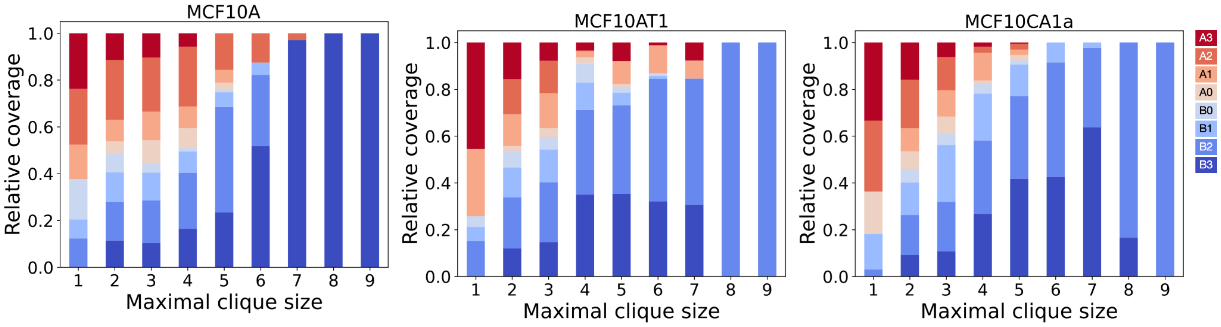

Fig. S24: Subcompartment composition of TAD cliques of size 1-9 in 10A (left), T1 (middle) and C1 (right). Cliques of size 1 and 2 are composed of singleton and binary TAD interactions not belonging to any clique.

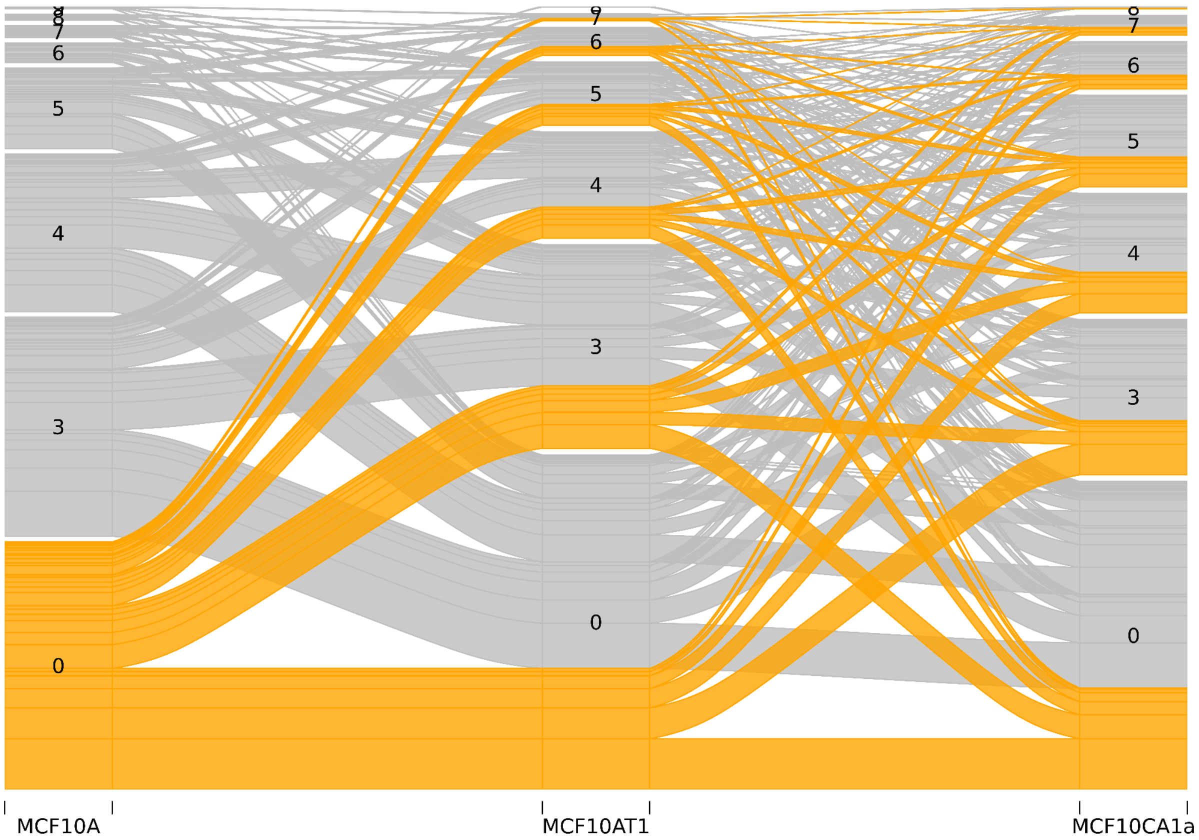

Fig. S25: Alluvial plot showing changes in TAD maximal clique size across the three cancer stages. The orange color highlights the alluvial path that starts with a non-clique (denoted as 0) in MCF10A (10A).

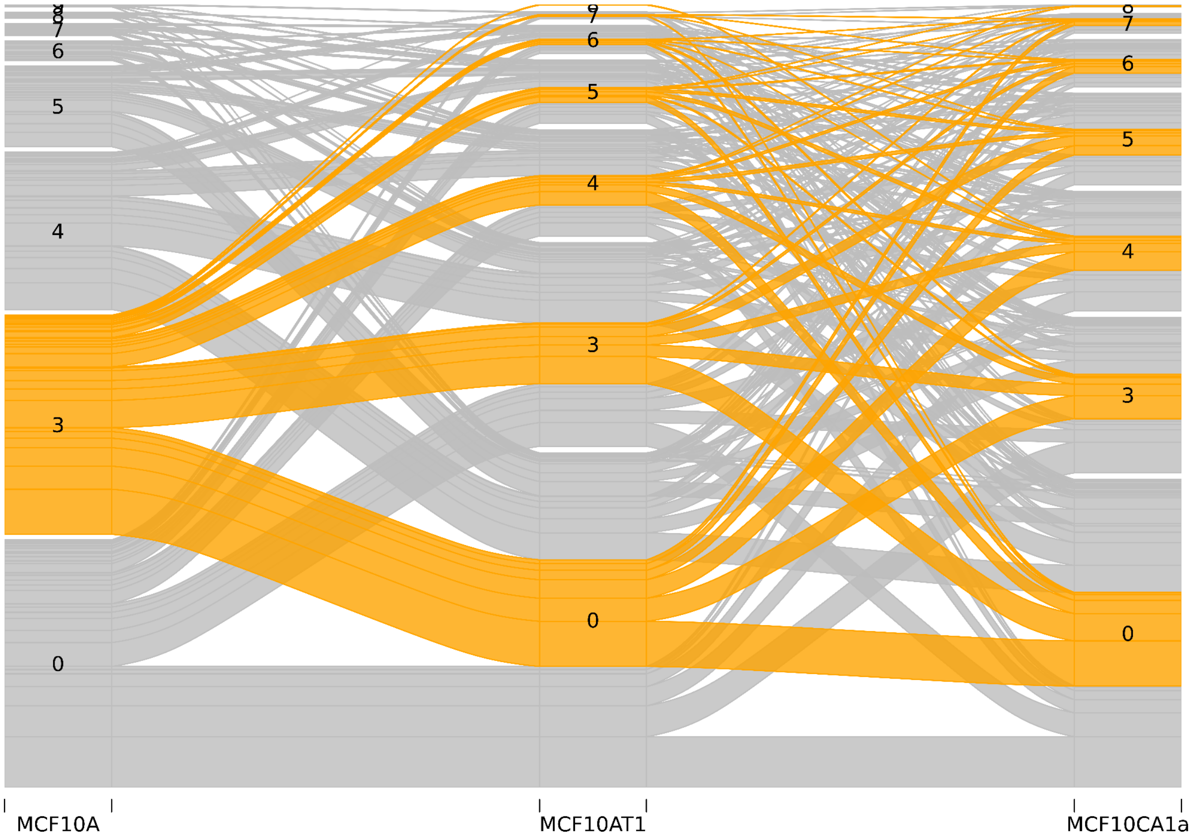

Fig. S26: Alluvial plot showing changes in TAD maximal clique size across the three cancer stages. The orange color highlights the alluvial path that starts with a clique of size 3 in MCF10A (10A).

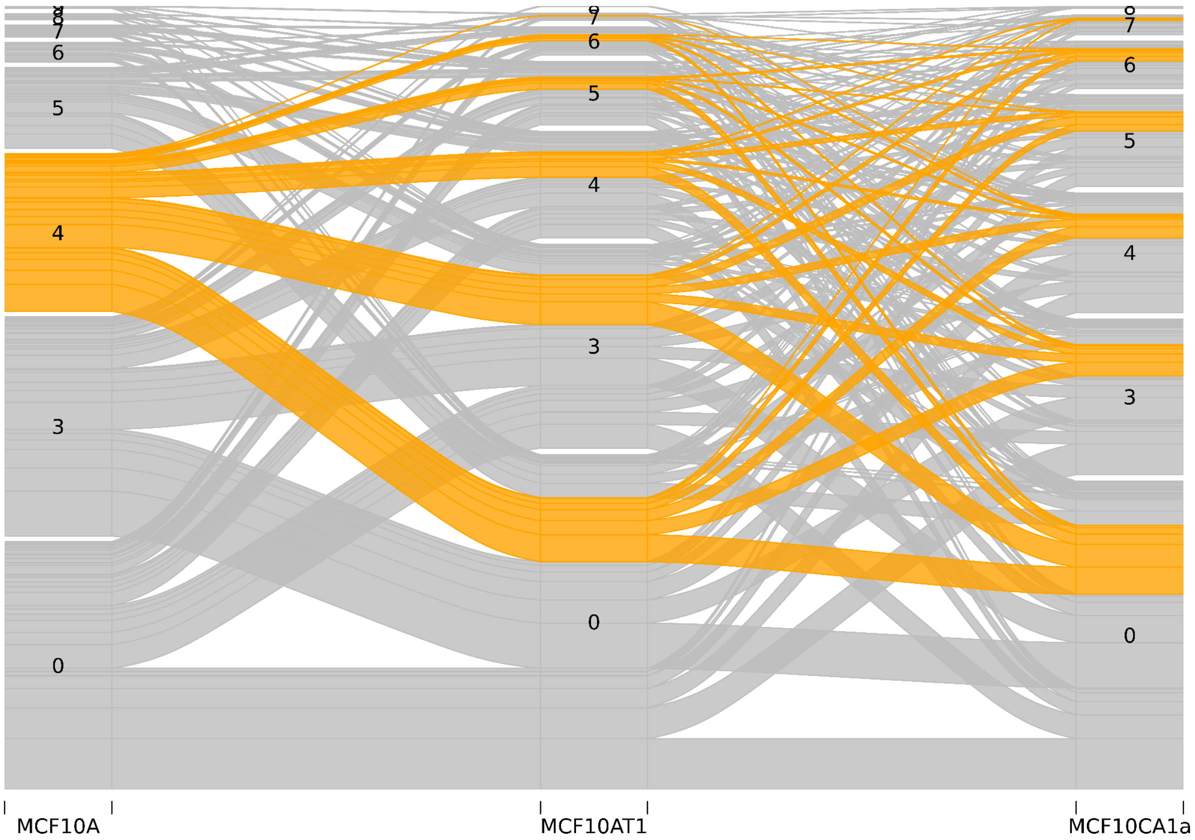

Fig. S27: Alluvial plot showing changes in TAD maximal clique size across the three cancer stages. The orange color highlights the alluvial path that starts with a clique of size 4 in MCF10A (10A).

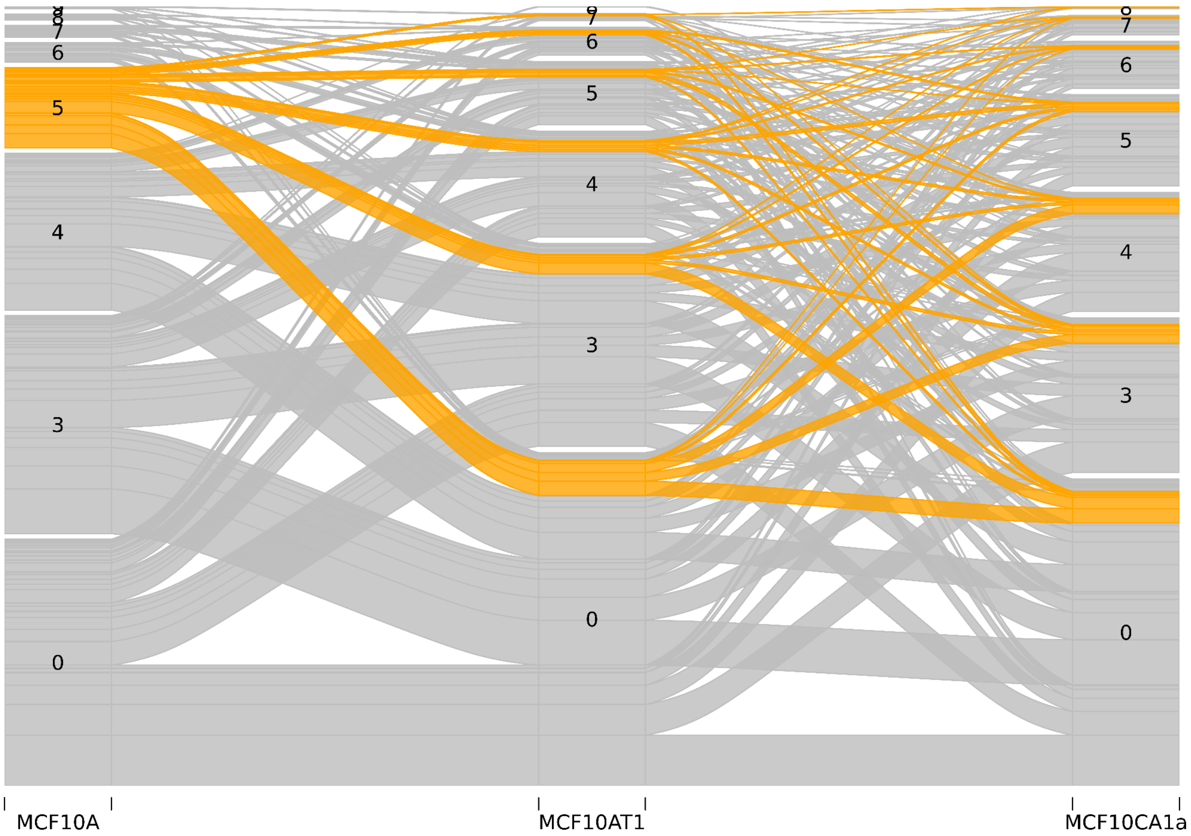

Fig. S28: Alluvial plot showing changes in TAD maximal clique size across the three cancer stages. The orange color highlights the alluvial path that starts with a clique of size 5 in MCF10A (10A).

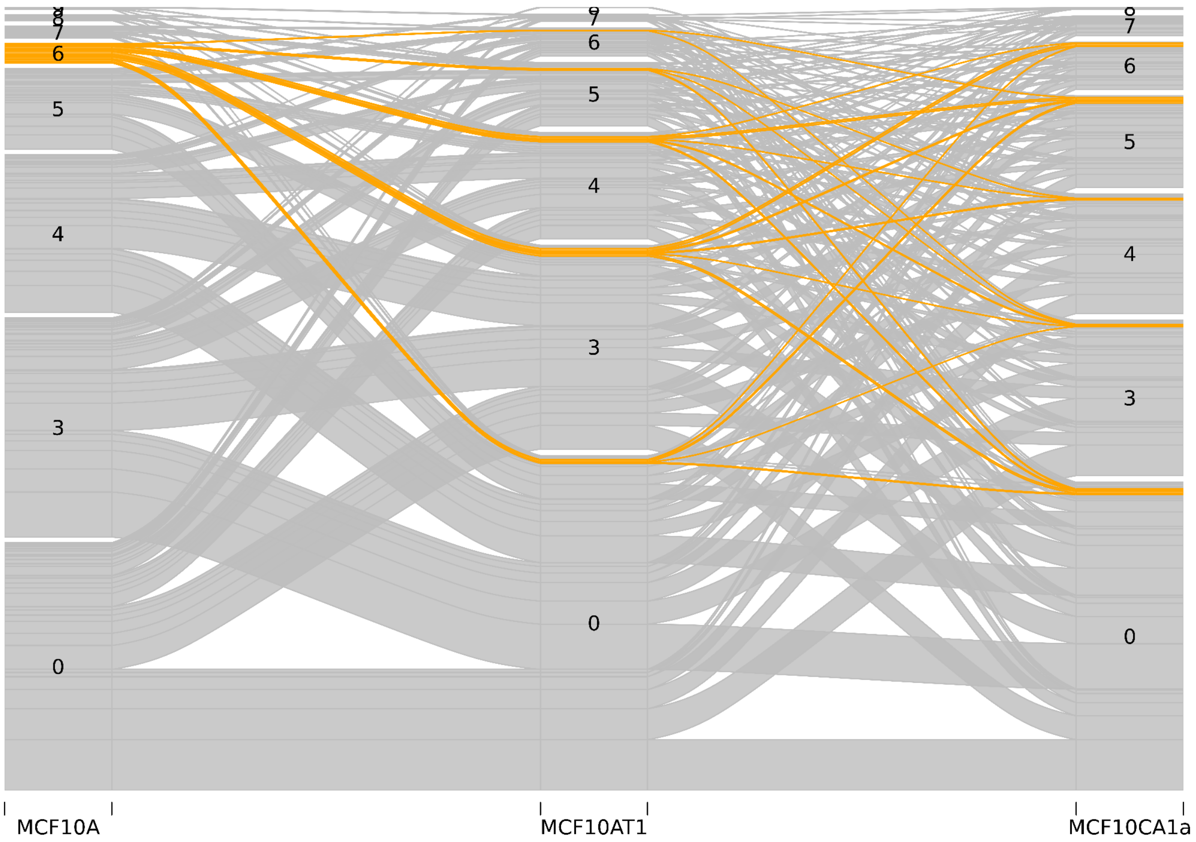

Fig. S29: Alluvial plot showing changes in TAD maximal clique size across the three cancer stages. The orange color highlights the alluvial path that starts with a clique of size 6 in MCF10A (10A).

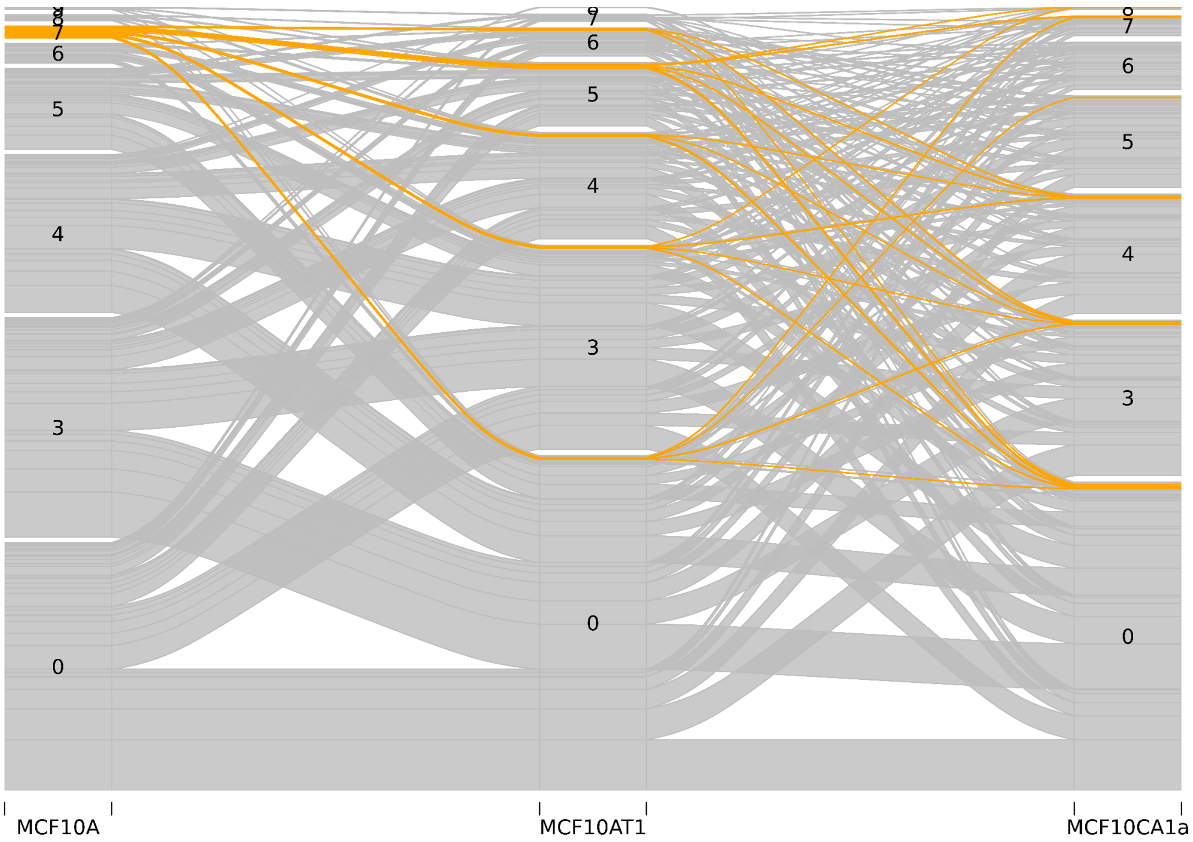

Fig. S30: Alluvial plot showing changes in TAD maximal clique size across the three cancer stages. The orange color highlights the alluvial path that starts with a clique of size 7 in MCF10A (10A).

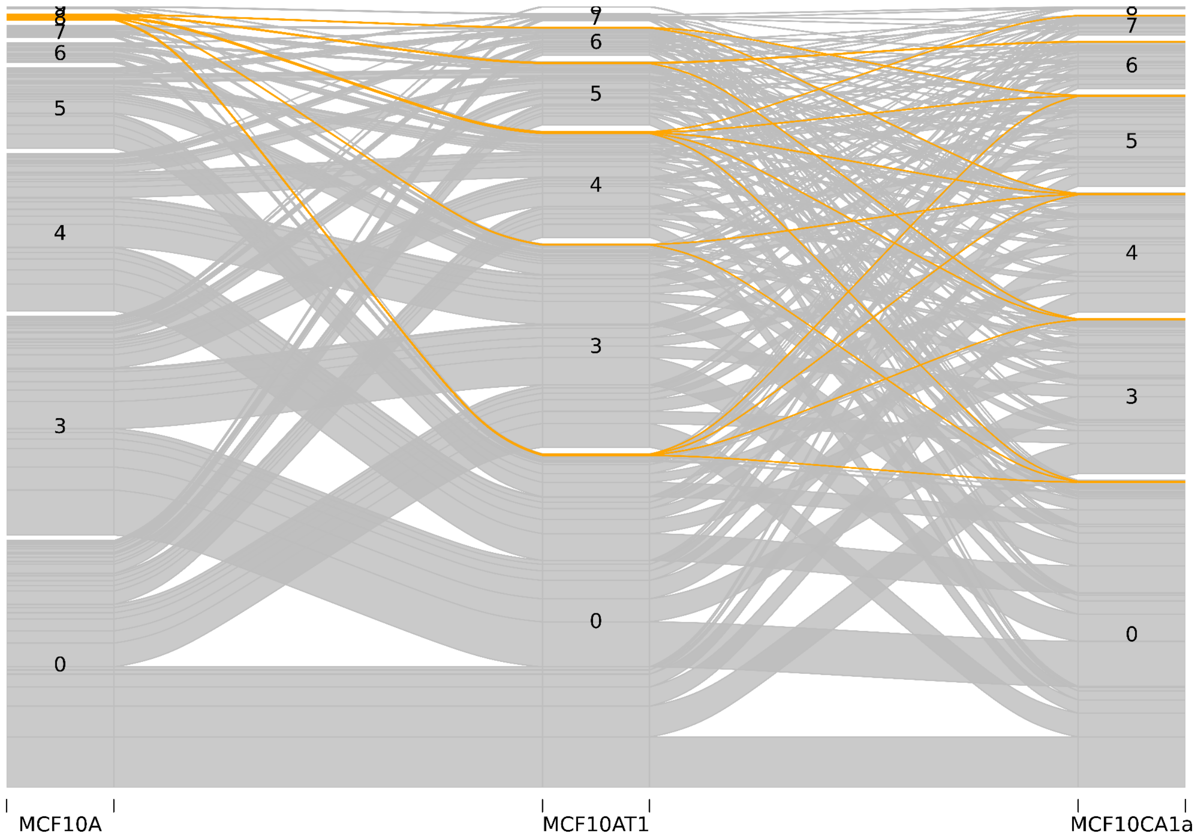

Fig. S31: Alluvial plot showing changes in TAD maximal clique size across the three cancer stages. The orange color highlights the alluvial path that starts with a clique of size 8 in MCF10A (10A).

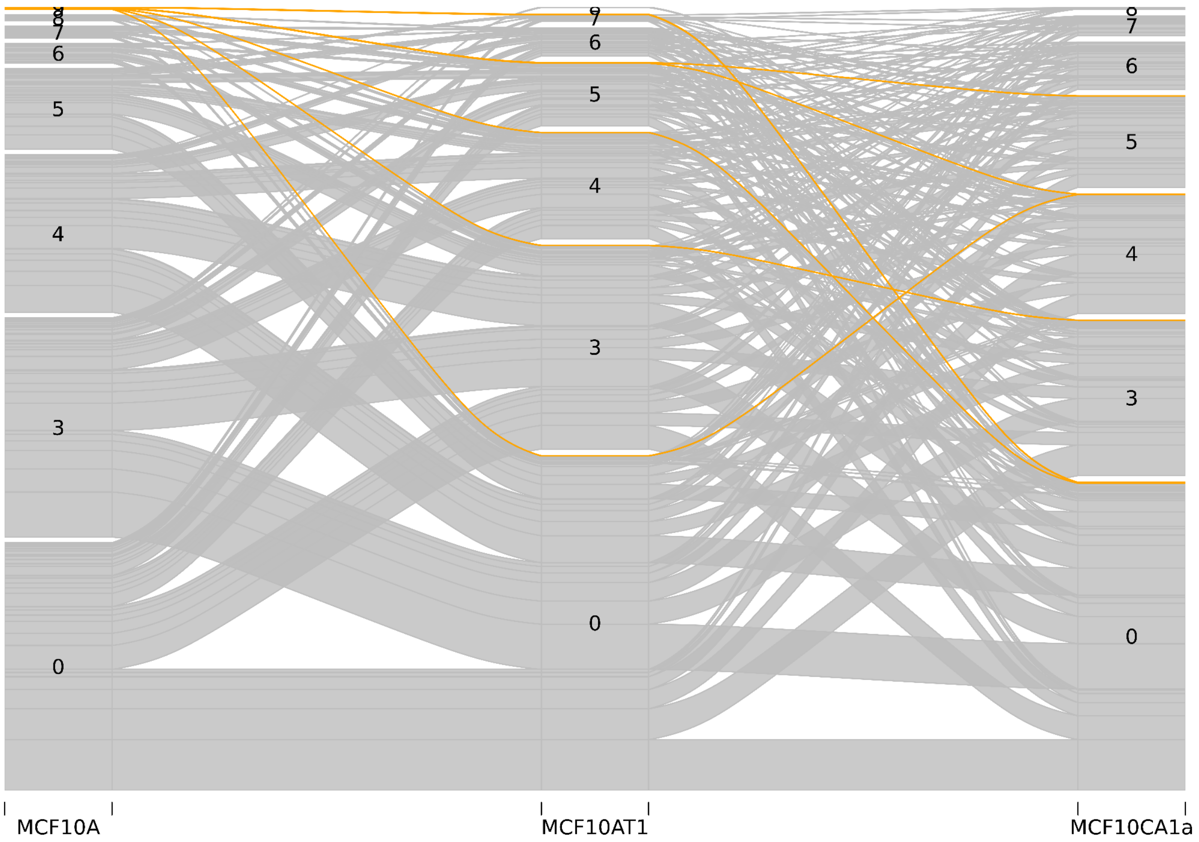

Fig. S32: Alluvial plot showing changes in TAD maximal clique size across the three cancer stages. The orange color highlights the alluvial path that starts with a clique of size 9 in MCF10A (10A).

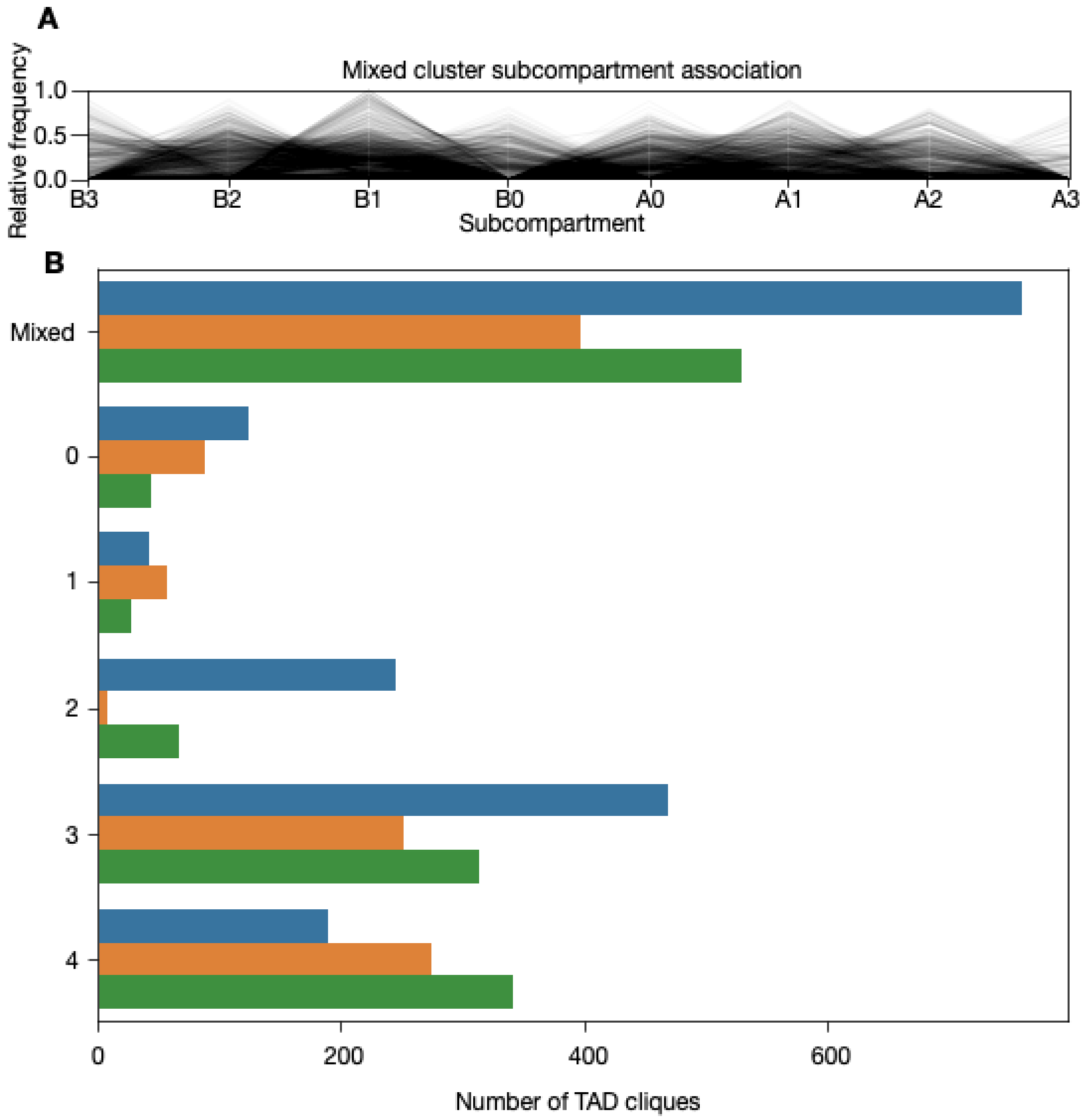

Fig. S33: A: Subcompartment enrichment for TAD cliques placed in the outlier ("mixed") cluster by HDBSCAN. **B**: Cluster sizes for TAD clique clusters shown in Fig. 4E (including the mixed cluster).

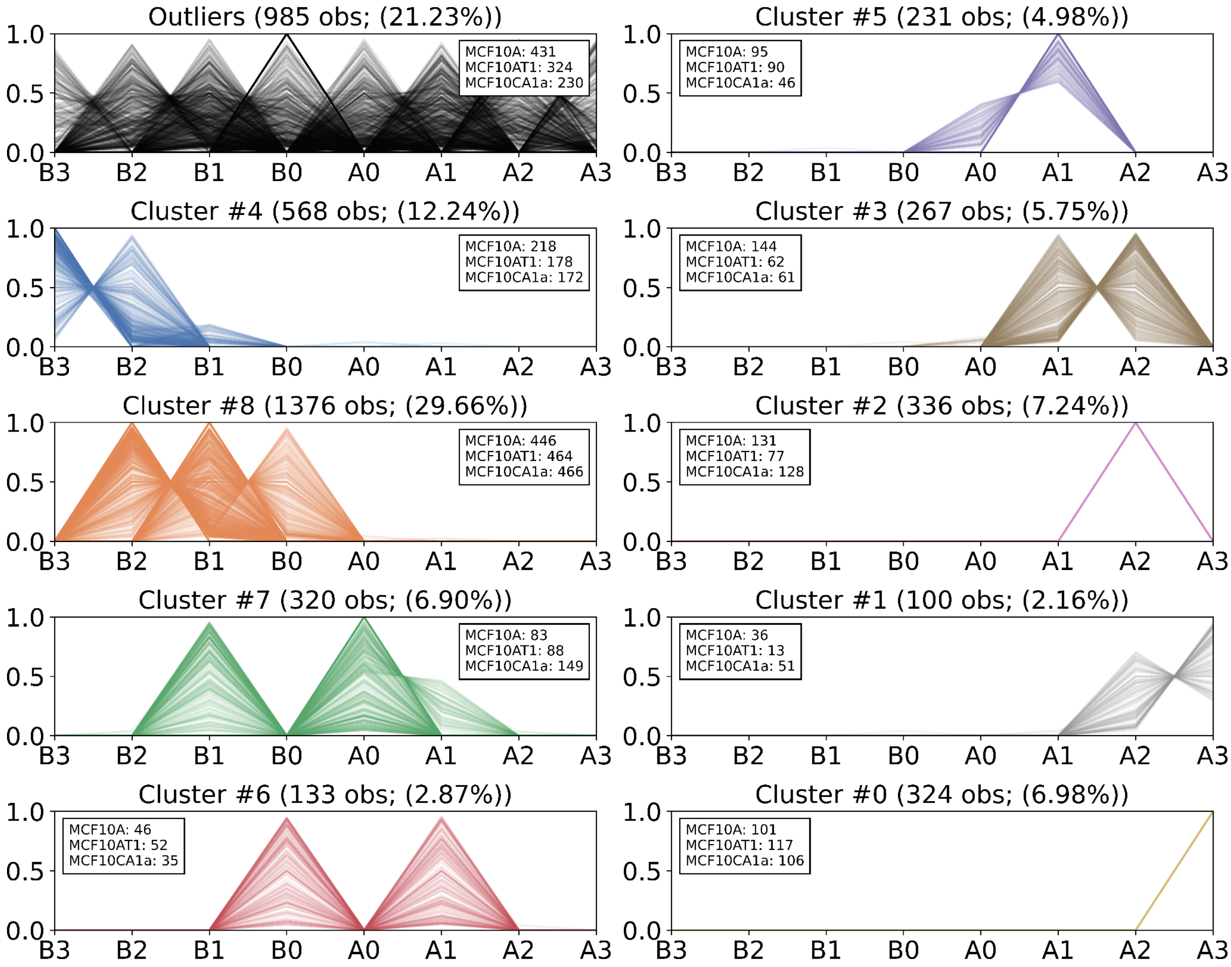

Fig. S34: Figure showing DBSCAN clustering analysis on TADs instead of cliques.

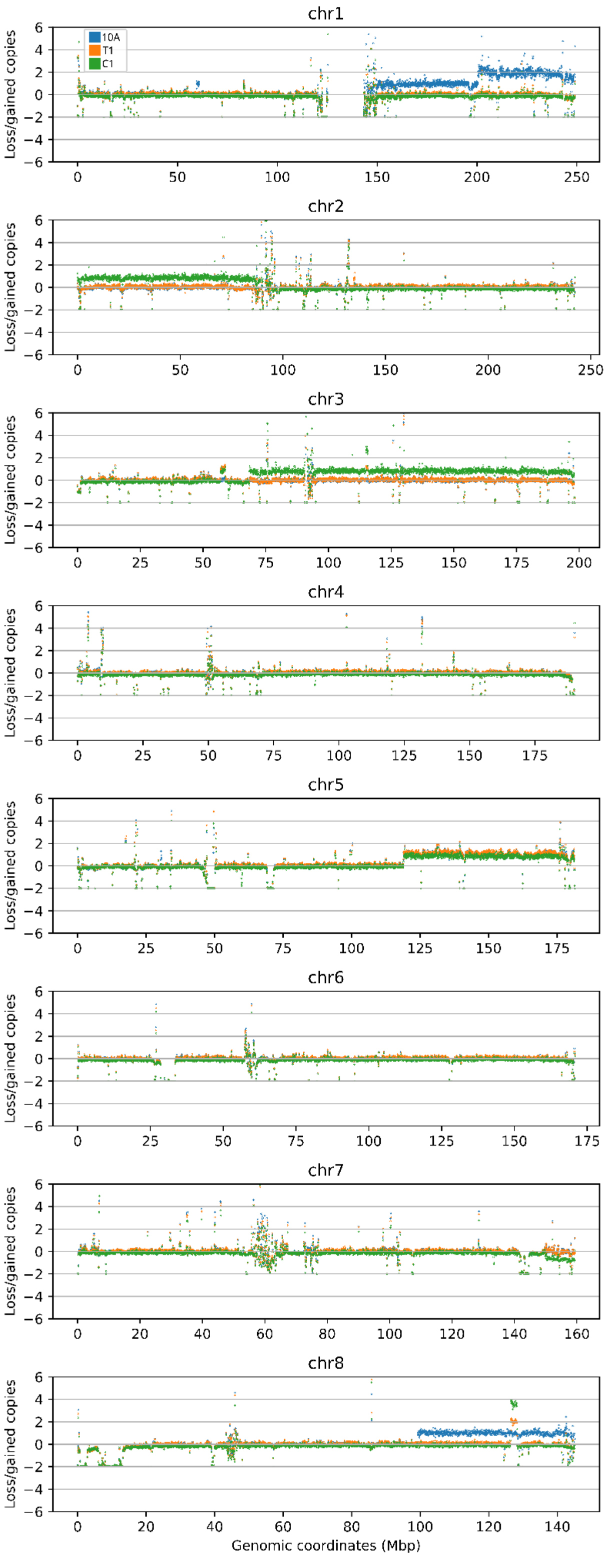

Fig. S35: Figure showing called CNVs across genome. Chrs 1-8

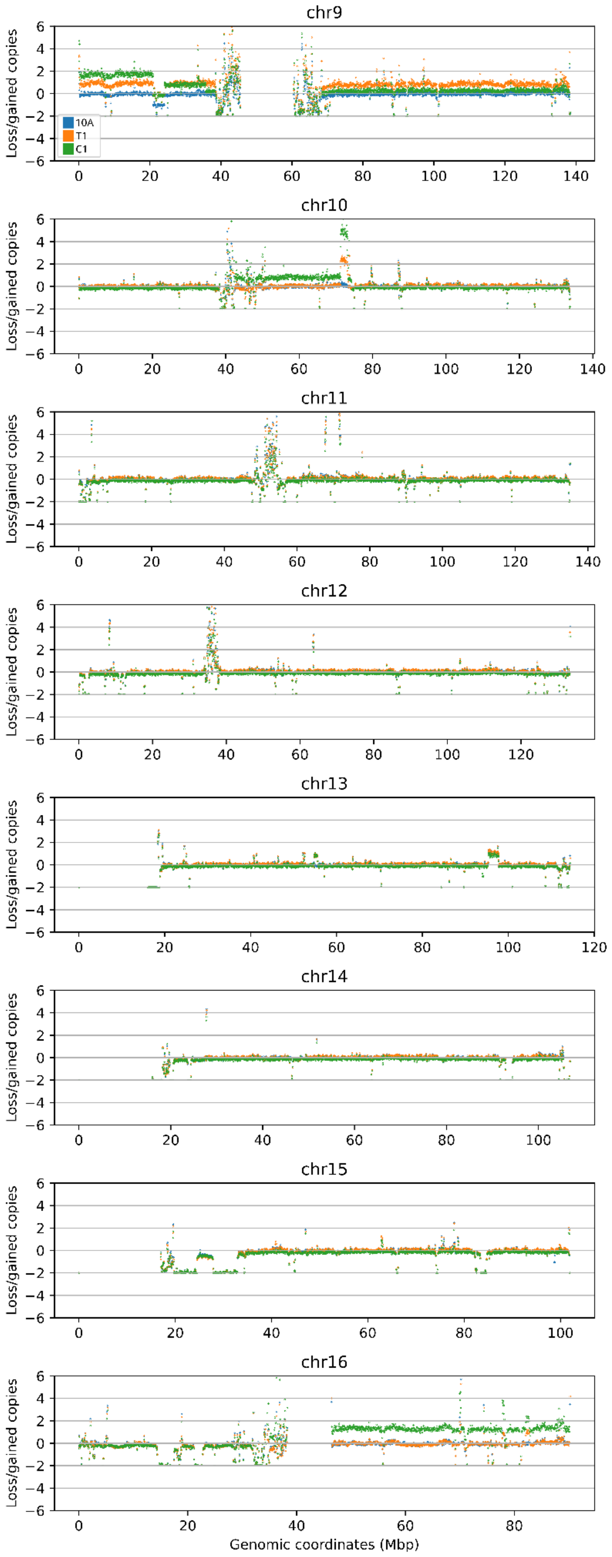

Fig. S36: Figure showing called CNVs across genome. Chrs 9-16

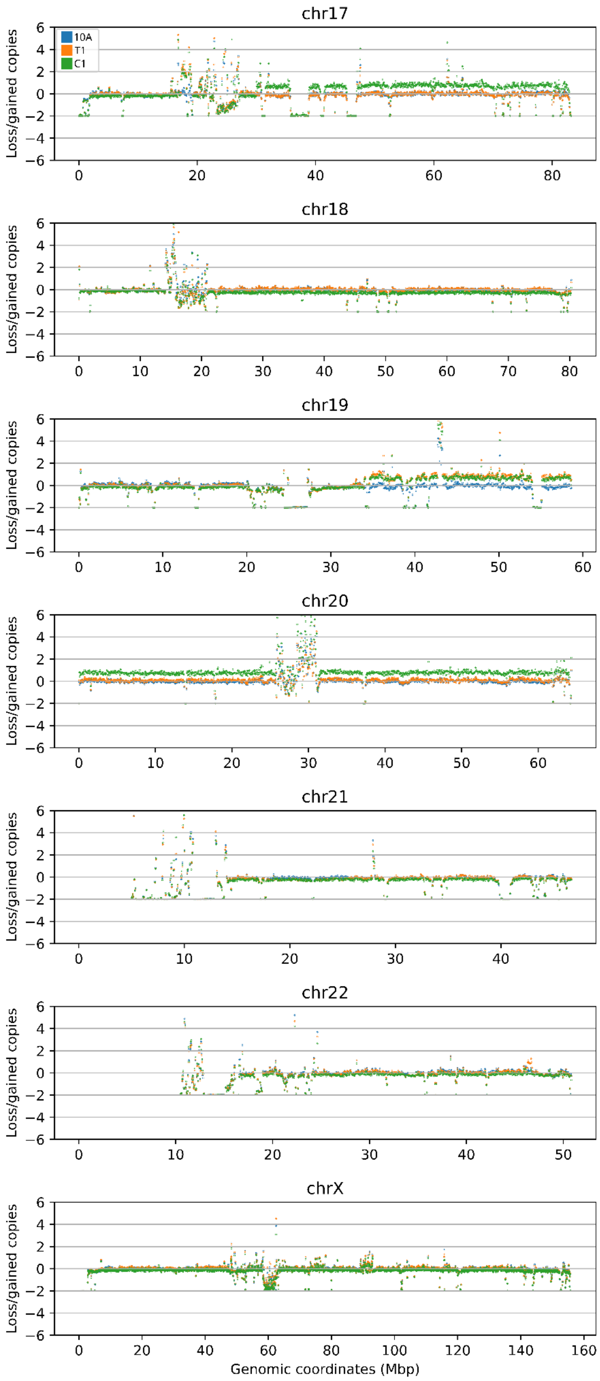

Fig. S37: Figure showing called CNVs across genome. Chrs. 17-X

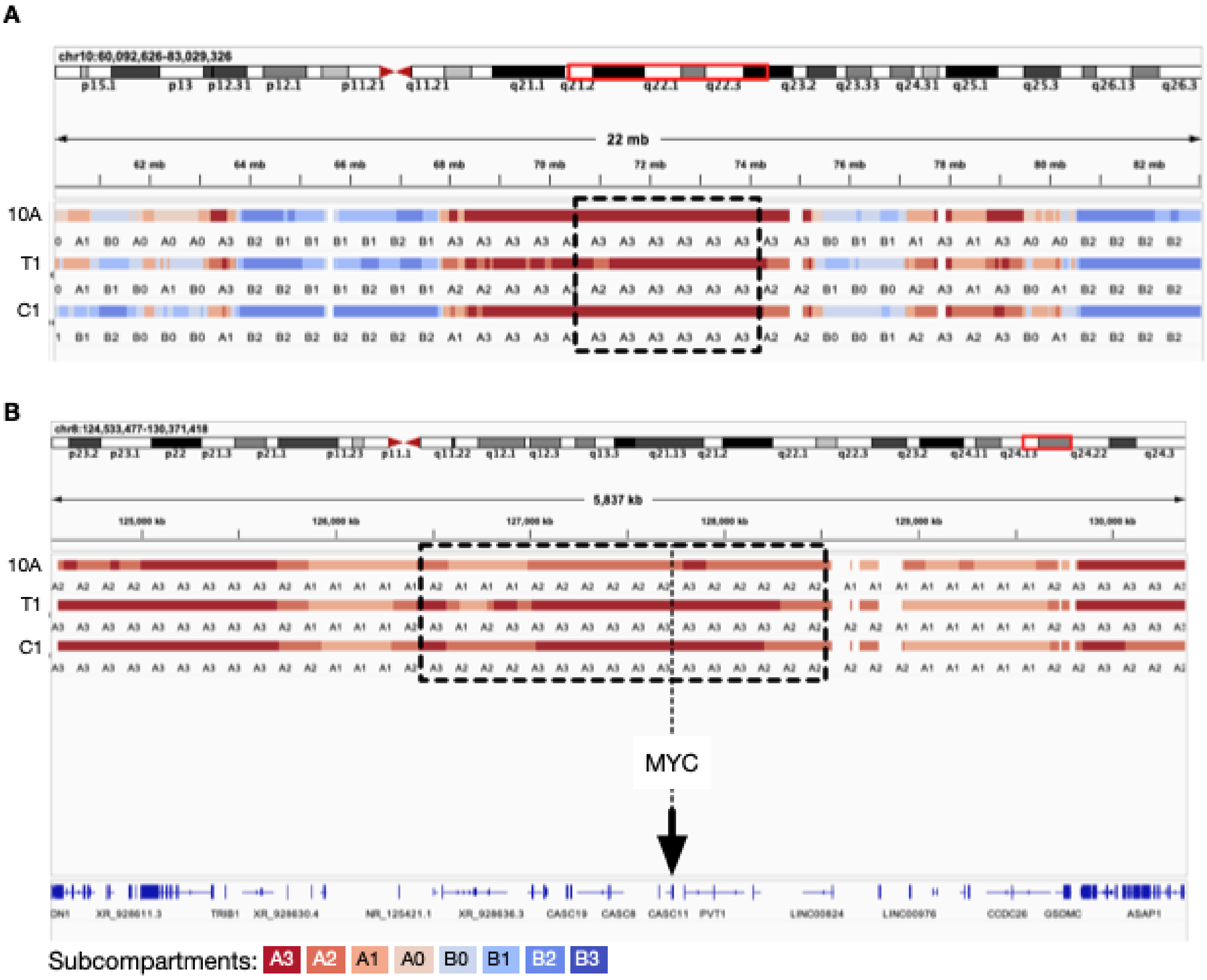

Fig S38: **A**: Genome browser view of subcompartments surrounding and including the amplified region in the three cell types on chromosome 10 (dotted lines) **B:** Genome browser view of subcompartments in the three cell types surrounding and including the amplified region on chromosome 10 (dotted lines)
