# Supplementary material for: Multi-level 3D genome organization deteriorates during breast cancer progression
Source: bioRxiv. 2023 Nov 27:2023.11.26.568711. Preprint. [Version 1] doi: 10.1101/2023.11.26.568711 (PMC10705249; doi:10.1101/2023.11.26.568711)
Supplement: Supplementary file 2 [file media-2.pdf]

Multi-level 3D genome organization deteriorates during breast cancer progression

Supplementary tables

Table S1: Hi-C interactions classified by type.

	MCF10A_R1	MCF10A_R2	MCF10AT1_R1	MCF10AT1_R2	MCF10ACA1a_R1	MCF10ACA1a_R2
reaported_pairs	694.20	815.52	837.73	718.09	672.54	786.82
valid_interaction	664.17	774.48	821.28	704.73	650.15	760.91
valid_interaction_rmdup	598.02	681.51	687.67	603.61	559.65	639.96
trans_interaction	95.93	109.50	112.73	101.96	135.70	158.56
cis_interaction	502.10	572.01	574.94	501.65	423.95	481.40
cis_shortRange	106.91	121.64	105.22	89.85	75.64	85.27
cis_longRange	395.19	450.37	469.72	411.80	348.31	396.13
cis_trans_ratio	5.23	5.22	5.10	4.92	3.12	3.04
cis_long_short_ratio	3.70	3.70	4.46	4.58	4.61	4.65

Table S2: Coverage of compartment switches.

MCF10A	MCF10AT1	MCF10CA1a	Coverage (Mbp)	Rel. coverage (%)
B	B	B	1024.22	37.58%
B	B	A	119.23	4.37%
B	A	B	76.1	2.79%
B	A	A	146.15	5.36%
A	B	B	101.57	3.73%
A	B	A	60.85	2.23%
A	A	B	97.91	3.59%

A	A	A	1099.61	40.34%

Table S3: Genome fraction (Mbp and relative) for WT, T1 and C1 for each of the 8 subcompartments.

**Subcompartment**	**Coverage (Mbp)**			**Coverage (relative)**		
	**MCF10A**	**MCF10AT1**	**MCF10CA1a**	**MCF10A**	**MCF10AT1**	**MCF10CA1a**
B3	164.82	180.49	172.04	5.34%	5.84%	5.57%
B2	509.71	449.93	468.55	16.50%	14.57%	15.17%
B1	517.25	581.01	580.49	16.75%	18.81%	18.80%
B0	355.62	268.17	251.65	11.52%	8.68%	8.15%
A0	213.53	253.13	216.38	6.91%	8.20%	7.01%
A1	546.21	538.50	482.78	17.69%	17.44%	15.63%
A2	398.25	438.56	523.43	12.90%	14.20%	16.95%
A3	382.88	378.47	392.95	12.40%	12.26%	12.72%

Table S4: Wilcoxon rank sum test results of the median subcompartment distance from the nucleus center in each Chrom3D simulation model per condition and subcompartment pair.

**Condition 1**	**Condition 2**	**Subcomp.**	**P-value**
10A	C1	A3	0.000209
10A	C1	A2	1.848e-12
10A	C1	A1	5.533e-06
10A	C1	A0	0.007654
10A	C1	B0	< 2.2e-16
10A	C1	B1	0.01355
10A	C1	B2	0.01856
10A	C1	B3	2.91e-12
10A	T1	A3	< 2.2e-16

10A	T1	A2	5.053e-13
10A	T1	A1	< 2.2e-16
10A	T1	A0	0.05495
10A	T1	B0	0.02693
10A	T1	B1	1.089e-12
10A	T1	B2	0.001588
10A	T1	B3	1.753e-12
C1	T1	A3	< 2.2e-16
C1	T1	A2	< 2.2e-16
C1	T1	A1	< 2.2e-16
C1	T1	A0	0.364
C1	T1	B0	< 2.2e-16
C1	T1	B1	0.00305
C1	T1	B2	0.7185
C1	T1	B3	0.8806

Table S5: Number of differentially expressed genes (lfc> 0.5; pvalue<0.001).

**contrast**	**condition**	**genes**	**DE genes**	**downreg**	**upreg**
10A	T1	21082	3180	1659	1521
T1	C1	21082	7510	3739	3771
10A	C1	21082	8362	4169	4193

Table S6: Table of numbers of members in HDBSCAN clusters in 10A, T1 and C1

**Cluster**	**MCF10A**	**MCF10AT1**	**MCF10CA1a**

	(10A)	(T1)	(C1)
Outliers	761	397	529
0	123	87	44
1	42	57	27
2	245	8	67
3	469	252	314
4	189	274	342
Total	1829	1075	1323

Table S7: Table of differentially expressed genes in T1 compared to 10A (lfc>= 0.1, svalue<0.001)

Gene name	chrom	start	end	log2FoldChange	svalue
LRATD2	chr8	126552443	126558478	1.53	4.32E-160
CASC19	chr8	127072694	127227541	-0.36	1.16E-06
CASC8	chr8	127289808	127482139	1.16	7.19E-24
ENSG00000286010	chr8	127663280	127670990	-1.32	0.00E+00
PVT1	chr8	127794526	128187101	0.28	2.95E-04
UNC5B	chr10	71212570	71302864	0.62	2.66E-09
SLC29A3	chr10	71319259	71381423	0.65	1.02E-05
VSIR	chr10	71747556	71773520	0.90	1.33E-26
PSAP	chr10	71816298	71851251	0.59	7.25E-12
CHST3	chr10	71964395	72013558	0.69	9.67E-15
SPOCK2	chr10	72059034	72089032	1.53	7.87E-06
ASCC1	chr10	72096032	72217134	1.15	5.94E-71
ANAPC16	chr10	72216000	72235860	1.09	1.19E-36
ENSG00000289506	chr10	72272288	72273704	2.36	6.49E-04
DDIT4	chr10	72273919	72276036	1.45	7.05E-127

DNAJB12	chr10	72332830	72355149	0.71	3.01E-36
MICU1	chr10	72367340	72626131	1.03	3.08E-59
MCU	chr10	72692143	72887694	1.36	2.49E-133
P4HA1	chr10	73007217	73096974	0.36	1.27E-05
NUDT13	chr10	73110375	73131828	1.96	7.12E-30
ENSG00000272599	chr10	73124573	73125532	1.45	4.49E-12
ECD	chr10	73130155	73169055	1.30	3.06E-105
FAM149B1	chr10	73168119	73244504	0.66	3.85E-21
ENSG00000288559	chr10	73247342	73248268	0.87	1.71E-05
MRPS16	chr10	73248843	73252693	0.42	2.03E-17
DNAJC9-AS1	chr10	73252791	73254349	1.40	3.09E-11

Table S8: Table of differentially expressed genes in C1 compared to 10A (lfc>= 0.1, svalue<0.001)

Gene name	chrom	start	end	log2FoldChange	svalue
LRATD2	chr8	126552443	126558478	2.09	1.62E-304
PCAT1	chr8	126556323	127419050	2.11	1.13E-17
CASC19	chr8	127072694	127227541	0.86	1.38E-36
ENSG00000224722	chr8	127086263	127087510	2.13	1.26E-05
ENSG00000287781	chr8	127253213	127257630	3.13	7.17E-08
CASC8	chr8	127289808	127482139	2.90	1.54E-150
POU5F1B	chr8	127322183	127420066	2.95	4.37E-53
ENSG00000286010	chr8	127663280	127670990	-0.41	1.98E-04
CASC11	chr8	127686343	127738987	1.92	1.45E-05
MYC	chr8	127735434	127742951	0.25	1.86E-04
PVT1	chr8	127794526	128187101	1.67	1.57E-122
UNC5B	chr10	71212570	71302864	3.43	1.44E-266

UNC5B-AS1	chr10	71217220	71218294	1.33	2.33E-04
SLC29A3	chr10	71319259	71381423	0.74	6.98E-07
CDH23	chr10	71396920	71815947	1.77	1.40E-04
VSIR	chr10	71747556	71773520	3.25	0.00E+00
PSAP	chr10	71816298	71851251	3.09	0.00E+00
ENSG00000289592	chr10	71888499	71889171	3.21	1.39E-10
CHST3	chr10	71964395	72013558	1.03	2.71E-32
SPOCK2	chr10	72059034	72089032	4.04	5.13E-29
ASCC1	chr10	72096032	72217134	2.03	8.81E-228
ANAPC16	chr10	72216000	72235860	1.22	4.50E-46
ENSG00000289506	chr10	72272288	72273704	6.13	8.21E-14
DDIT4	chr10	72273919	72276036	2.14	6.95E-284
DNAJB12	chr10	72332830	72355149	0.77	5.06E-43
MICU1	chr10	72367340	72626131	1.09	2.50E-64
MCU	chr10	72692143	72887694	2.08	0.00E+00
P4HA1	chr10	73007217	73096974	2.29	6.14E-203
NUDT13	chr10	73110375	73131828	2.63	1.71E-51
ECD	chr10	73130155	73169055	2.22	0.00E+00
FAM149B1	chr10	73168119	73244504	1.78	8.33E-158
DNAJC9	chr10	73183362	73247255	0.46	2.33E-12
ENSG00000288559	chr10	73247342	73248268	1.94	6.42E-19
MRPS16	chr10	73248843	73252693	0.50	2.83E-25
DNAJC9-AS1	chr10	73252791	73254349	1.87	5.05E-18

Table S9: List of manually annotated translocations for 10A.

**chrom1**	**start1**	**end1**	**chrom2**	**start2**	**end2**

chr3	894000	68025000	chr9	23591000	138394717
chr3	1235000	68434000	chr5	118992000	181538259
chr3	68025000	198295559	chr9	0	20878000
chr5	118960000	181538259	chr9	23908000	138394717

Table S10: List of manually annotated translocations for T1.

**chrom1**	**start1**	**end1**	**chrom2**	**start2**	**end2**
chr3	0	59225000	chr17	16340000	83257441
chr3	894000	68025000	chr9	23591000	138394717
chr3	1235000	68434000	chr5	118992000	181538259
chr3	56933000	90650000	chr17	0	19300000
chr3	68025000	198295559	chr9	0	20878000
chr5	118960000	181538259	chr9	23908000	138394717
chr6	0	170805979	chr19	33715000	58617616
chr8	126000000	128300000	chr10	0	133797422

Table S11: List of manually annotated translocations for C1.

**chrom1**	**start1**	**end1**	**chrom2**	**start2**	**end2**
chr2	0	92100000	chr10	41600000	133797422
chr3	0	59225000	chr17	16340000	83257441
chr3	0	68462000	chr7	0	150000000
chr3	894000	68025000	chr9	23591000	138394717
chr3	1235000	68434000	chr5	118992000	181538259
chr3	56933000	90650000	chr17	0	19300000
chr3	68025000	198295559	chr9	0	20878000
chr5	118960000	181538259	chr9	23908000	138394717
chr6	0	170805979	chr19	33715000	58617616
chr7	63700000	151700000	chr9	24123000	35780000

chr8	126000000	128300000	chr10	0	133797422
chr10	0	74500000	chr17	29970000	83257441
